# Visual Feature Learning on Video Object and Human Action Detection: A Systematic Review

**DOI:** 10.3390/mi13010072

**Published:** 2021-12-31

**Authors:** Dengshan Li, Rujing Wang, Peng Chen, Chengjun Xie, Qiong Zhou, Xiufang Jia

**Affiliations:** 1 Institute of Intelligent Machines, Hefei Institutes of Physical Science, Chinese Academy of Sciences, Hefei 230031, China; dengshan@mail.ustc.edu.cn (D.L.); cjxie@iim.ac.cn (C.X.); zhouqiong@ahau.edu.cn (Q.Z.); xfjia@iim.ac.cn (X.J.); 2 Science Island Branch of Graduate School, University of Science and Technology of China, Hefei 230026, China; 3 Intelligent Agriculture Engineering Laboratory of Anhui Province, Hefei 230031, China; 4 School of Computer Science and Technology, Anhui University, Hefei 230601, China

**Keywords:** video object detection, human action recognition, deep learning, temporal information, optical flow, LSTM, video dataset

## Abstract

Video object and human action detection are applied in many fields, such as video surveillance, face recognition, etc. Video object detection includes object classification and object location within the frame. Human action recognition is the detection of human actions. Usually, video detection is more challenging than image detection, since video frames are often more blurry than images. Moreover, video detection often has other difficulties, such as video defocus, motion blur, part occlusion, etc. Nowadays, the video detection technology is able to implement real-time detection, or high-accurate detection of blurry video frames. In this paper, various video object and human action detection approaches are reviewed and discussed, many of them have performed state-of-the-art results. We mainly review and discuss the classic video detection methods with supervised learning. In addition, the frequently-used video object detection and human action recognition datasets are reviewed. Finally, a summarization of the video detection is represented, e.g., the video object and human action detection methods could be classified into frame-by-frame (frame-based) detection, extracting-key-frame detection and using-temporal-information detection; the methods of utilizing temporal information of adjacent video frames are mainly the optical flow method, Long Short-Term Memory and convolution among adjacent frames.

## 1. Introduction

### 1.1. Background and Motivation

Video object detection and human action recognition are applied to various scenarios, such as the recognition of vehicle plate numbers in traffic monitoring systems, the detection of dangerous vehicle behaviors, the detection of running red lights, the detection of abnormal production behaviors in industrial production, the identification of abnormal passenger behaviors at stations and airports, etc.

The difficulties of video detection include video defocus, motion blur, part occlusion, etc. Video defocus would be generated during the focusing process. The defocus of the video and the motion of the object may cause the video defocus and motion blur. Occlusion between objects may cause the part occlusion. In addition, the shape of the objects in the video may be changing with the distance of the camera. Therefore, compared with image detection, video detection should be more challenging.

The existing video detection methods are operated on frames. Most of the existing video detection methods are to decompose the video into frames, and then use the image detection method to detect the frames. Therefore, the speed of video detection depends on the speed of image detection. In addition, some methods directly operate on the video, however, these methods are also frame-based. They operate adjacent frames by using specific algorithms. Therefore, for video detection, image detection methods are still important.

Before, the methods applied to image detection include Histogram of Oriented Gradients (HOG) [1], Scale-Invariant Feature Transform (SIFT) [2], Haar-like feature [3], etc. Haar-like feature is from Haar wavelet [4], which is a kind of square-shaped function. These above methods are used to extract the features of the image, and then used for detection. The HOG method first grayscales the image, and then performs Gamma correction [5] for reducing the impact of local shadows and lighting changes in the image, and it can also suppress the noise interference. HOG captures the outline of the object, computes the gradient histogram of each cell in the image, and combine the gradient histogram of each cell to generate the descriptor. SIFT searches key points in different scales and calculates the direction of the key points. The key points will not change when the illumination, affine transformation and noise of images changes. The key points include corner points, edge points, bright spots in dark areas, and dark spots in bright areas, etc. The advantages of SIFT include good stability, good feature distinctiveness, high feature recognition rate. HAAR uses the feature template sliding in the image, calculates the feature value, and recognizes the image by a classifier.

Machine learning classifiers include Support Vector Machine (SVM) [6], Random Forest [7], and some loss functions, etc. SVM maps data to space, and classifies the data through a hyperplane. The advantage of SVM is high classification accuracy, the disadvantage may be large computational consumption and large storage space.

Random Forest is based on Decision Tree [8]. Decision Tree is the classifier that simulates human. Decision Tree selects the class which has more votes. Random Forest is the multiple Decision Trees. Random Forest usually consist of hundreds to thousands of Decision Trees. After training, Random Forest classifiers can often achieve high accuracy.

In the deep learning tasks, some loss functions can also be regarded as the classifiers, since only the class which conforms to the loss function can be detected and recognized. These loss functions include cross-entropy function [9] and some loss functions customized by researchers themselves.

Before deep learning, local feature extraction methods such as SIFT, HOG, etc., did not have the ability of feature translation invariance. The possible reason may be that the features extracted by these methods may be simpler than the deep learning methods.

The detection speed of one-stage detector is usually faster than two-stage detector. The two-stage detector has higher detection accuracy, but the detection speed would be reduced. The two-stage detector usually follows the two steps: extracting features from the input (feature extractor), recognizing the features by the trained classifiers (classifier). The difference between one-stage detector and two-stage detector is mainly that the two-stage detector often has a separate feature extractor, which is called “backbone” in some literatures. Meanwhile, the one-stage detector combines the feature extractor and classifier into one, which can reduce the complexity of the network structure and improve the detection speed, but the detection accuracy may be reduced.

Generally, video object and human action detection can be classified into three categories: detecting frames by the image detector, extracting the key frames for the detection, or using temporal information between adjacent frames. The former is implemented frame by frame, and the speed of video detection depends on the speed of the frame detection. Moreover, these methods usually do not extract the key frames from videos. Thus, the base of these methods is still image detection. Some algorithms, such as You Only Look Once (YOLO) [10], use the structure of Feature Pyramid Networks (FPN) [11]. Feature pyramid networks (FPN) is used in one-stage detectors such as YOLO, and two-stage detectors such as Faster Regions with Convolutional Neural Networks Features (Faster R-CNN) [12], Mask R-CNN [13], Residual Net (ResNet) [14], etc.

The latter of the above paragraph includes optical flow [15] and Long Short-Term Memory (LSTM) [16]. Optical flow is to aggregate the feature maps of adjacent frames and to improve the detection accuracy of blurry frames. Some other methods are similar to optical flow, such as using convolutions to aggregate the feature maps of adjacent frames. Many video detection algorithms use the architecture of LSTM or modified LSTM into their own structure.

The connection between object tracking and object detection is the method of object tracking could be used for object detection, since object tracking and object detection both use the temporal information of the video frames.

### 1.2. Contributions

The contribution of this paper is summarized as follows:

(A) Review the commonly-used video-based detection datasets, and their application scope.

(B) Review the machine learning-based models, which are used for video object and human action detection.

(C) Summarize and analyze the performance of the classical video detection algorithms, and summarize the methods of improving the speed of video detection.

(D) Summarize and analyze the image and video evaluation metrics used in the literatures, and illustrate that most video metrics use image detection metrics by frames.

(E) Summarize the algorithms of video surveillance system, face detection, face recognition, face tracking, image and video quality enhancement, respectively.

(F) Summarize the three main ideas for video detection: the first is to detect each frame; the second is to extract the key frames; the third is to adopt the LSTM structure, the optical flow method or convolution among adjacent frames for using the temporal information among adjacent frames.

### 1.3. Paper Organization

The main structure of the paper is: first, the video datasets are introduced; second, the video detection algorithms are introduced and analyzed; third, the video detection algorithms are discussed and evaluated. Among them, the video detection algorithms are classified as the frame-by-frame algorithms (Section 3) and the using-temporal-information algorithms (Section 4).

**Figure 1:**
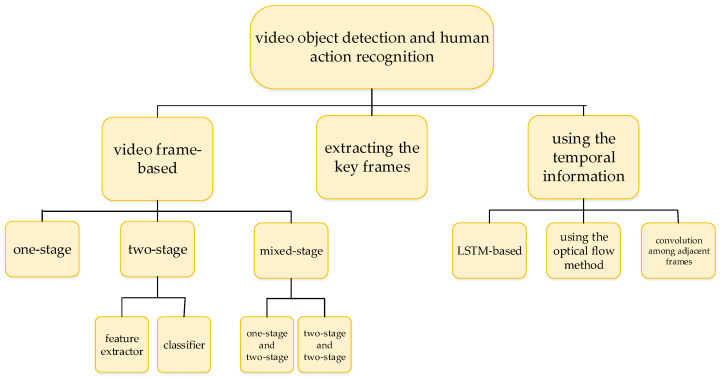
The summarization of video object and human action detection. The paper is organized as this structure as well.

The paper reviews the video object detection and human action recognition using deep learning methods, summarizes the current video detection approaches. Our paper is organized as follows: Section 2 summarizes the image detection metrics and the most commonly used video classification datasets. Section 3 describes the frame-based (frame-by-frame) video object detection approaches. Section 4 describes the video detection methods by extracting the key frames. Section 5 analyses the video object and human action detection approaches which use the temporal information. Section 6 discusses and analyzes the performance of video detection algorithms and remarks the limitations and future research directions of the reviewed methods. Section 7 remarks the limitations of the current algorithms, and discusses the future research directions, in our own opinion. Finally, Section 8 gives a conclusion about the video object and human action detection. The summarization of the video object and human action detection is shown in Figure 1.

## 2. Machine Learning-Based Evaluation Metrics and Video-Based Datasets

### 2.1. Machine Learning-Based Evaluation Metrics

The machine learning-based evaluation metrics used for image detection mainly include: *accuracy*, *precision*, *recall*, *F1-score*, *AP*, *mAP*, ROC, AUC, etc. These metrics are from the classification results of the positive and the negative data. The positive data indicates the correct data, and the negative data indicates the wrong data. The calculation factors of these metrics are: True Positive (*TP*), True Negative (*TN*), False Positive (*FP*), False Negative (*FN*). *TP* represents the number of the positive classes predicted as the positive classes. *TN* represents the number of the negative classes predicted as the negative classes. *FP* represents the number of the negative classes predicted as the positive classes. *FN* represents the number of the positive classes predicted as the negative classes.

(1) $$accuracy=\frac{TP+TN}{TP+TN+FP+FN}$$

(2) $$precision=\frac{TP}{TP+FP}$$

(3) $$recall=\frac{TP}{TP+FN}$$

(4) $$F1=\frac{2\times precision\times recall}{precision+recall}$$

(5) $$AP=\frac{\sum_{i=1}^{N}precision}{N}$$

(6) $$mAP=\frac{\sum_{i=1}^{M}AP}{M}$$

**Figure 2:**
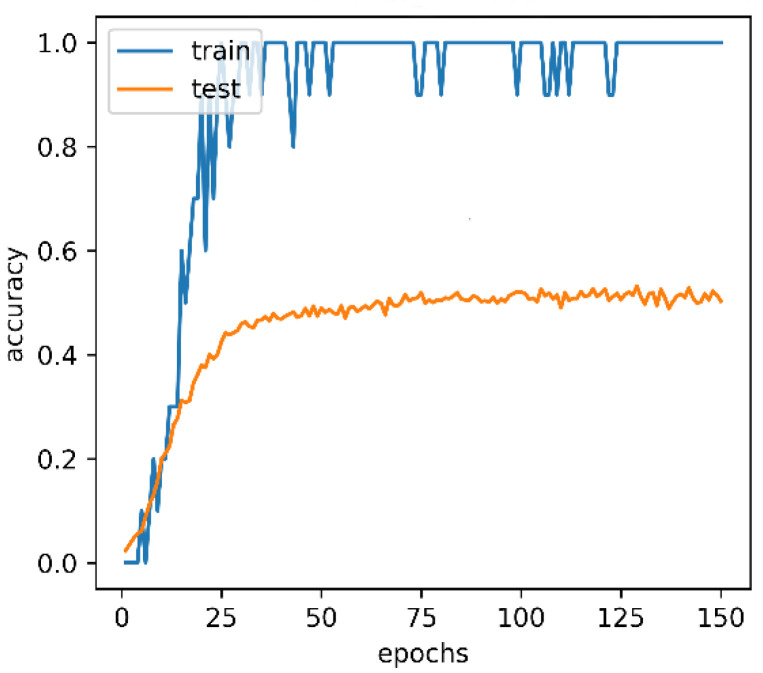
The training accuracy and the test accuracy of a CNN-LSTM model, which was implemented in nearly 3 days. The dataset is UCF101. The accuracy is an important metric on UCF101. The training accuracy is the result of the training data on the model. The test accuracy is the result of the test data on the model. The metric of accuracy is using image detection metrics by frames.

Receiver Operating Characteristic (ROC) curve uses the False Positive rate and the True Positive rate as the coordinate axes. The area under the ROC curve is Area Under Curve (AUC). Figure 2 illustrates the training accuracy and the test accuracy of a CNN-LSTM model on UCF101 [17] dataset.

From the existing video detection literatures, it could be found that most of the video detection evaluation use the image metrics. The method is to use video frames for calculation. We think that there are many differences between video and image. Video frames are always not independent of each other, but images are independent of each other. Thus, using image metrics to the video frames may cause the duplicated statistics, such as TP, FP, etc., since the same objects in the frames may be counted repeatedly.

### 2.2. Video-Based Datasets for Object Detection and Action Recognition

The commonly used video classification datasets are as follows: ImageNet VID dataset [18], which has 3862 snippets for training, 555 snippets for validation, 937 snippets for test. The dataset has 30 classes. These classes are carefully selected as considering different factors, such as motion type, background interference, average number of objects, etc. Each frame of the videos is annotated. Another video object detection dataset is YouTube-Objects dataset [19], which is collected from YouTube, has 10 object classes. The videos in the dataset are formed as the frames, these frames can be restored to videos if necessary. A video object dataset that has man-made bounding boxes is YouTube-BoundingBoxes dataset [20], which contains 380,000 19-s-long videos, with 23 classes of objects. The quality of the video is similar to that of a mobile phone. The project is made by Google Brain, and the dataset has 5.6 million human-annotated bounding boxes. A video object detection dataset used for urban geographic recognition is Apolloscape dataset [21], which provided by Baidu includes RGB videos with high-resolution images and per-pixel annotations. The dataset defines 26 different objects, such as cars, bicycles, pedestrians, buildings, street lights, etc. CDnet2014 [22] is a video change detection dataset, which has 11 categories.

Some video datasets are used for video segmentation, which is based on video object detection. These video datasets include: Cambridge-driving Labeled Video Database (CamVid) [23], which is the dataset of video semantic segmentation. The data was taken from the perspective of driving cars, has 32 semantic classes. Densely Annotated Video Segmentation [24] is an object segmentation dataset with high-definition video, including 50 videos, 3455 annotated frames, and the video resolution is 1080p. DAVIS dataset [25] is used for video segmentation, which has 50 clips, 3455 annotated frames.

Some video datasets are used for pedestrian and human face detection. These datasets include: UCSD Pedestrian dataset [26] is a video dataset containing pedestrians, which can be used for computer vision tasks such as pedestrian detection and recognition. Caltech Pedestrian dataset is also a dataset for pedestrian detection. Similarly, there are ETH Pedestrian dataset, INRIA Pedestrian dataset, TudBrussels Pedestrian dataset, Daimler Pedestrian Dataset. DeeperForensics-1.0 [27], containing 60,000 human face videos, which is used to train models, for the purpose of detecting forgery faces.

Video tracking is based on video object detection either. Amsterdam Library of Ordinary Videos for tracking (ALOV++) [28] is an object tracking video dataset, which is used to detect and track similar objects under different light, transparency and focal length. The videos are from YouTube, with an average duration of 9.2 s. VOT dataset [29] is for VOT-Challenge, which is also a video object tracking dataset. MOT dataset [30] is a video multi object tracking dataset, used for MOT-Challenge.

Human action recognition uses the video object detectors. The datasets include: HMDB51 [31], which is a dataset published in a paper on behavior recognition. There are 51 human action categories, 6766 video clips. The video clips are classified as facial actions and body actions. UCF101 [17] is an action and sports recognition dataset. The dataset has 101 human action categories, 13320 videos. The dataset is from YouTube. ASLAN [32] is an action recognition dataset, which has 432 action categories and 3697 video clips. Sports-1M [33], an action and sports recognition dataset, has 487 action categories and 1,100,000 video clips. FCVID [34] is a human action and activity recognition dataset, scene and objects recognition. There is 239 action categories and 91,223 video clips in the dataset. ActivityNet [35], is an action recognition and human activity dataset. Youtube-8M [36] is an action recognition video dataset, which has 3862 action categories and 5,600,000 video clips. Charades [37] is a human action and activity recognition dataset, which has 157 action categories and 9848 video clips. Kinectics [38] is a human action recognition dataset, has 600 action categories and 500,000 video clips. The scale of Kinectics is larger than UCF101. AVA [39] is a human action recognition dataset, and has 50,000 video clips from YouTube. VLOG [40] is an action recognition dataset, and has 114,000 video clips. HACS [41] is an action recognition and action localization dataset. The dataset has 200 action categories and 520,000 video clips. 20BN-SOMETHING-SOMETHING [42] is a human action recognition dataset. The dataset has 174 action categories and 220847 video clips.

## 3. Video Frame-Based Object Detection Algorithms

Most video detection methods decompose the video into frames, use the image detection model to detect. Therefore, almost all image detectors can be applied for video detection. The other video detection methods utilize the correlation between frames, operate on adjacent frames. Some of the methods which operate on adjacent frames use LSTM-like models. The following discusses in detail.

### 3.1. One-Stage Video Object Detection

The current object detection methods are divided into two categories, one-stage object detection and two-stage object detection. In the two-stage object detection, feature extraction is the first stage, the classification is the second stage. One-stage object detection methods include YOLO [10], SSD [43] and RetinaNet [44]. Their common point is that the detection speed of a single frame is very fast, and real-time video detection can be implemented.

#### 3.1.1. You Only Look Once (YOLO)

YOLO [10] makes the object classification as “regression”. In the training, YOLO will resize the images to a specific size, which can be set in the program. In the model, the nonlinear mapping between image features and neural network parameters is established. In the detection, the image or video is performed.

**Figure 3:**
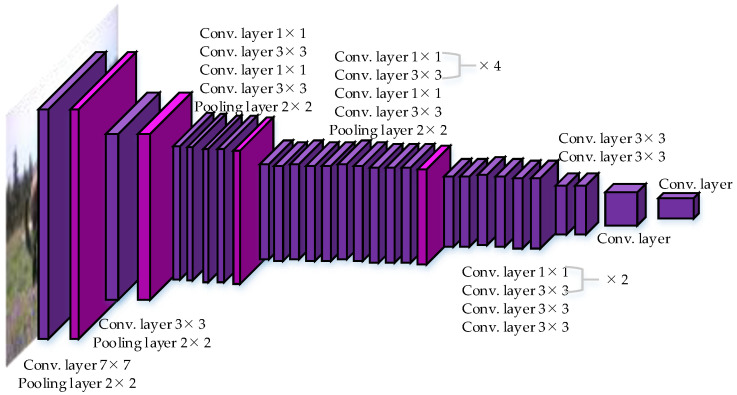
The network structure of YOLO, the purple ones are convolutional layers, the purple-red ones are max pooling layers. The structure has 26 convolutional layers and 4 max pooling layers. The layers are for extracting features of the objects, the rear layers are for classification of the objects. The design of the structure should have some fixed routines. The planes reflect the size of the feature maps. The dimension of the network is not shown in the figure. The video frame shown in the figure is from the YouTube Objects (YTO) dataset [19].

The network structure of YOLO uses the structure of GoogLeNet [45] for classification, and replaces the inception modules of GoogLeNet with 1 × 1 and 3 × 3 convolutional layers, in order to simplify the structure and improve the detection speed. YOLO has 24 convolutional layers and 2 fully connected layers. The network structure is illustrated in Figure 3.

YOLO divides the input image into s × s grid cells, each grid cell only predicts one object, but an object may occupy multiple cells and may be predicted by multiple cells. Every grid cell generates B (number) bounding boxes, and the bounding box like the anchor box used in Faster R-CNN, has several different scales and aspect ratio. If an object occupies multiple grids, the bounding boxes of the grids merge into one. The bounding box is not only used to positioning, but also used to generate the object confidence. Each bounding box has 5 parameters: x, y, w, h, and the confidence. (x, y) denotes the center coordinate of the bounding box, w and h denote the relative location to the whole image. The detection confidence value is defined as follow:

(7) $$confidence=\mathrm{Pr}\left(Object\right)\times IoU_{pred}^{truth}$$

where Pr(*Object*) is the probability of the object, *IoU* represents Intersection-over-Union, which is the quotient of the intersection and union of the candidate bounding box and the ground truth bounding box. The Pr(*Object*) is 1 when an object locates at a grid cell, or else the value is 0. The second item of the right side of the above equation is the *IoU* value, which is the overlapping area of the bounding box and the ground truth.

In the test, the bounding boxes are filtered by Non-Maximum Suppression (NMS). Compared with the previous video frame-based object detection methods, such as the frame detection methods using R-CNN, Faster R-CNN, Deformable Parts Model (DPM) [46] etc., YOLO has such advantages:

(A) The detection speed of YOLO is very fast. As YOLO does not have the separate stage of generating the region proposals, the detection speed of YOLO is 45 FPS using TITAN X GPU, and the speed of Fast YOLO can reach 155 FPS with the same type of GPU. Since the playback speed of the video is about 30 frames per second, the real-time video detection can be implemented when the video detection speed reaches 30 FPS. Compared with the previous real-time detection system, such as Deformable Parts Model (DPM) [46], YOLO’s mean Average Precision (mAP) value has been increased by more than two times.

(B) YOLO uses the context information to enhance the detection accuracy. Since YOLO does not limit the number of grids occupied by the object, then, the relationship between the grids is relatively close. In the two-stage object detection, the classifier only detects the pixels in the region proposals, while YOLO uses the context information more. Thus, the detection accuracy is higher.

(C) YOLO can learn high semantic features. As shown in Figure 3, YOLO’s network structure is deep enough so that the features are advanced and easy to classify. In addition, YOLO performs a lot of optimization, thus, the network is fast and the features are generalized.

#### 3.1.2. YOLO9000 (YOLOv2)

YOLOv2 [47] uses a series of methods to improve detection accuracy and speed, and adopts strategies to enable YOLOv2 to detect more than 9000 objects. In addition, the basic framework of YOLOv2 is similar to YOLOv1.

YOLOv2 uses the following methods to improve the detection speed: (A) YOLOv2 adopts Darknet19 as the detection neural network, which has 19 convolutional layers with 3 × 3 filter and 5 max pooling layers with doubling the number of channels compared with the previous layer. (B) YOLOv2 follows almost every 3 × 3 convolution layer with a 1 × 1 convolution layer, which may reduce the complexity of network computing and improve the detection speed. (C) YOLOv2 does not use the dropout layer, which may reduce the network computational complexity and help increase the network speed.

In addition, the following methods are used to improve the detection accuracy: (A) YOLOv2 uses Batch Normalization (BN) [48] after every convolutional layer, which could improve the detection accuracy, by unifying the distribution of all data to the standard normal distribution. (B) YOLOv2 uses data augmentation, which can randomly crop and rotate the input image, which is equivalent to expanding the input dataset, so that the established model contains more features. (C) YOLOv2 improves the resolution of the input image, increases the detected pixels, and increases the amount of detected information, which is conducive to improving the detection accuracy. (D) YOLOv2 removes the fully connected layer and uses anchor box to predict the bounding box directly. (E) YOLOv2 adds an identity mapping which is similar to residual skip connection, reduces the information loss caused by convolution and pooling, and improves the detection accuracy.

The structure of DarkNet19 used in YOLOv2 is shown in Table 1. As YOLOv2 adopts a strategy of constructing dataset, which is the method WordTree, it can detect more than 9000 categories of objects. Common datasets may not have a tree type, and the WordTree method constructs labels of dataset as a tree, the probability of a leaf node is the product of the parent nodes. YOLO uses COCO dataset [49] to detect, and uses ImageNet dataset to classify.

#### 3.1.3. YOLOv3

YOLOv3 [50] still uses the framework of DarkNet, and the network uses the residual module and the multi-scale prediction. The multi-scale prediction is similar to Feature Pyramid Networks (FPN) [11]. Compared with YOLOv2, YOLOv3 uses more residual skip modules presented in ResNet [14], which reduces the loss of the information caused by convolution and pooling, making the network deeper, which can extract more advanced semantic features and improve the recognition accuracy.

**Figure 4:**
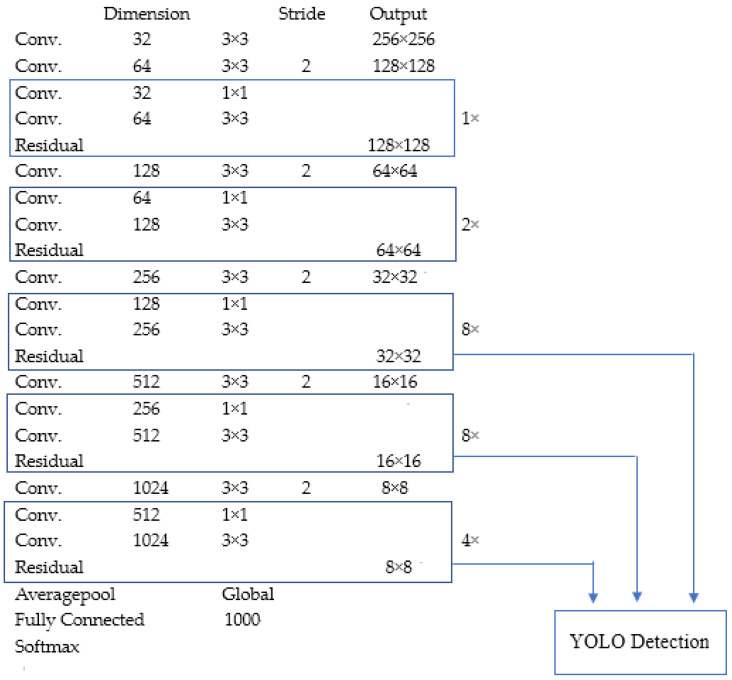
The network structure of YOLOv3. YOLOv3 uses the idea of Feature Pyramid Networks. Small-size feature maps are used to detect large-size objects, and large-size feature maps are used to detect small-size objects. YOLOv3 concatenates the output feature maps of 32 × 32, 16 × 16, 8 × 8 for detection.

YOLOv3 uses multi-scale prediction to enhance the detection accuracy. In DarkNet53, the final detection result is synthesized by Scale1, Scale2 and Scale3, which is illustrated in Figure 4. YOLOv3 does not use the fully connected layer either, which reduces the complexity of network computing and improves the detection speed. Because of the above methods, it ensures the detection accuracy. The detection accuracy of DarkNet53 is as much as ResNet152 [14], but the detection speed is much higher than ResNet152.

YOLOv3 uses convolution instead of pooling for down-sampling, which reduces the information loss in the neural network iteration. Usually, the information loss of pooling may be large, because the pooling operation merges multiple pixels into one pixel.

#### 3.1.4. YOLOv4

The detection speed and detection accuracy of YOLOv4 [51] are improved, compared with YOLOv3. YOLOv4 has three parts: backbone, neck and head. The backbone is used for extracting features. The neck is used for transmitting the extracted features to the part of head. The head is used for object classification and bounding box regression.

YOLOv4 uses Cross Stage Partial Networks (CSP Darknet) [52] as the backbone. CSPNet solves the problem of gradient information duplication in other backbones, and integrates the gradient changes into the feature map, therefore, YOLOv4 reduces the parameter amount and FLOPS of the model, improves the detection speed and accuracy, and reduces the size of the model. CSPNet is based on the idea of DenseNet. CSPNet uses the shortcut connections for reducing the information loss in the transmission, effectively alleviates the gradient disappearance.

YOLOv4 uses PANet [53] as the neck. The neck can generate the feature pyramids. PANet is based on Mask R-CNN [13] and FPN [11]. The neck adopts a kind of FPN structure that enhances the bottom-up transmission, which improves the transmission of the bottom features.

**Figure 5:**
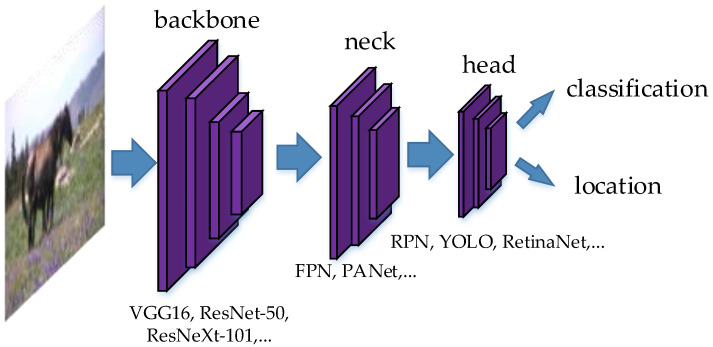
The structure of YOLOv4, which has 3 parts: backbone, neck and head. The backbone is used to extract the features of the object, the neck is used to transmit the features, the head is a detector, which classifies the object in the image (frame), and indicates the location of the object in the image (frame). Each part is constructed by the convolutional layers and pooling layers.

YOLOv4 uses the YOLOv3 detector as the head. The characteristic of the head is fast detection speed and high detection accuracy. In the head, each object class generates three kinds of anchor boxes, corresponding to the three different object scales and sizes. The structure of YOLOv4 is shown in Figure 5.

#### 3.1.5. Using-Dilated-Convolution Unmanned Aerial Vehicle (UAV) Detection

**Figure 6:**
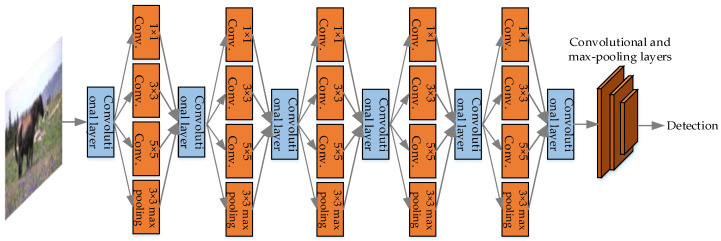
The structure of using-dilated-convolution UAV detection, which has 5 Inception modules.

Yavariabdi et al. [54] propose a framework which is based on YOLOv3 tiny, for UAV detection. The structure improves the backbone of YOLOv3 tiny, adds the Inception module which is from GoogLeNet, and adds the dilated convolutions. The model uses 5 Inception modules, and the dilated factor is 2. In the detection, the scalable kernel correlation filter (sKCF) is integrated into the model to improve the detection speed. Generalized Intersection over Union loss is used in the system. The experimental results show that the UAV video detection framework improves the detection accuracy, and the detection speed is not reduced. The structure is shown in Figure 6.

#### 3.1.6. FastUAV-NET

**Figure 7:**
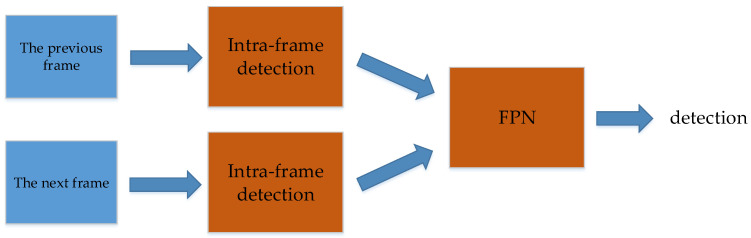
The workflow of FastUAV-NET.

Yavariabdi et al. [55] also propose a UAV detection method called FastUAV-NET, which is an improvement of [54]. FastUAV-NET uses intra-frame detection and inter-frame tracking. Intra-frame detection is similar to the literature [54], which also uses 5 Inception modules. The inter-frame tracking between frames uses the structure of a Feature Pyramid Network (FPN). The proposed model achieves a good trade-off between detection speed and detection accuracy. The workflow is shown in Figure 7.

#### 3.1.7. Single Shot MultiBox Detector (SSD) and Other Improved Versions

Single Shot MultiBox Detector (SSD) [43] uses the anchor of Faster R-CNN [12], and performs the multi-scale prediction, i.e., generates the multi-scale feature map to obtain a nonlinear mapping between the image and the features. It makes the detection speed of SSD fast, and the localization of bounding box is as accurate as YOLOv2.

In the multi-scale feature maps of SSD, the large feature map is used to detect small objects, and the small feature map is used to detect large objects. Since the information loss of the large feature map is little.

The loss function of SSD has two parts: the loss that calculates the detection confidence of the object, and the loss that calculates the location of the object. As shown in Equation (8).

(8) $$L\left(x,c,l,g\right)=\frac{1}{N}\left(L_{conf}\left(x,c\right)+\alpha L_{loc}\left(x,l,g\right)\right)$$

where *N* is the number of boxes, which is mapped to Ground Truth, and the parameter α is the ratio between the confidence loss and the location loss, the default value of α is 1. Here *x* evaluates how much the *i*-th detection bounding box matches the *j*-th ground truth bounding box, and $x_{ij}^{p}\in \left\{0,1\right\}$. When $x_{ij}^{p}=1$, it means that the *i*-th detection box matches the *j*-th ground truth, and the class of ground truth is *p*. The parameter *c* is the value of class confidence. The parameter *l* is the location value of the detection box, and *g* is the position value of the ground truth.

The location regression loss uses Smooth L1 loss function, which is one of the classic regression functions. The confidence loss is a classic softmax loss function. The location regression loss and the confidence loss are shown in Equations (9) and (10).

(9) $$L_{loc}\left(x,l,g\right)=\overset{N}{\underset{i\in Pos}{\sum }}\underset{m\in \left\{cx,cy,w,h\right\}}{\sum }x_{ij}^{k}smooth_{L1}\left(l_{i}^{m}-{\hat{g}}_{j}^{m}\right)$$

(10) $$L_{conf}\left(x,c\right)=-\overset{N}{\underset{i\in Pos}{\sum }}x_{ij}^{P}\log\left({\hat{c}}_{i}^{P}\right)-\underset{i\in Neg}{\sum }\log\left({\hat{c}}_{i}^{0}\right)$$

where ${\hat{c}}_{i}^{P}=\frac{\exp\left(c_{i}^{P}\right)}{\sum_{P}\exp\left(c_{i}^{P}\right)}$, *x, l, g, c, N* are the same with Equation (8), *m* is the pixel within a bounding box. *Pos* denotes the positive samples of the bounding boxes, *Neg* denotes the negative samples of the bounding boxes, *smooth*_*L*1_ is the Smooth L1 loss function.

The detection speed of SSD is fast, and the detection accuracy is similar or higher than Faster R-CNN [12]. SSD also uses the data augmentation [56], such as image flipping, image cropping, image distortion, etc. Data augmentation has a significant effect on improving the mAP value. Because the detection speed of SSD is fast, about 59 FPS, SSD can be used for real-time video object detection.

**Figure 8:**
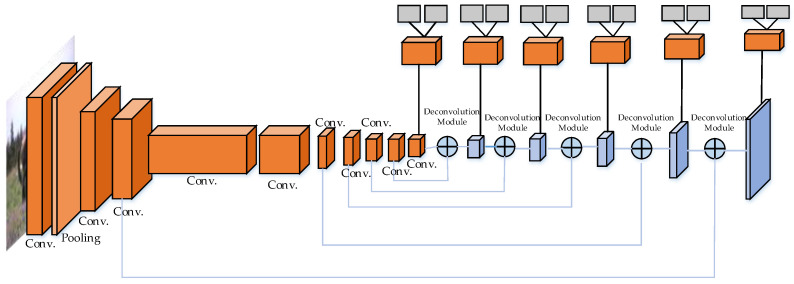
The structure of DSSD. The orange boxes are convolutional layers, the light-orange boxes are pooling layers, the lilac boxes are de-convolutional layers, the lilac gates represent the concatenation of the convolution and the deconvolution. There are 10 convolutional layers, 1 pooling layer and 5 de-convolutional layers in the main pipeline. The planes reflect the size of the feature maps, the thickness reflects the dimension of the feature map. The six branches on the top right of the figure represent the prediction module, i.e., classification and object localization module.

Compared with SSD, a difference of DSSD [57] is the addition of contextual information. DSSD replaces VGG-16 [58] with ResNet-101 [14], and uses the de-convolutional layers and skip connections to enhance the small object detection of the initial large feature map. Like SSD, the ResNet-101 module in DSSD also adopts the mode of multi-scale prediction. The feature maps are extracted from the front, middle, and back of ResNet-101, and summarized to the de-convolutional layer at the end of DSSD. The use of de-convolutional layers is another characteristic of DSSD. The aim of using the de-convolutional layer is to utilize the context information, which is beneficial for the detection of small objects via shallow feature maps. The structure is illustrated in Figure 8.

Other improved versions of SSD include Rainbow SSD (RSSD) [59], Feature Fusion Single Shot Multibox Detector (FSSD) [60], etc. RSSD does not replace the basic network VGG-16 in SSD with ResNet-101, but improves the feature concatenation method. In this way, shallow features and deep features are better used. Although the detection speed is reduced, the detection mAP value is improved.

RSSD improves SSD algorithm from the following two aspects: (A) RSSD uses the classification networks to strengthen the connection of feature maps between different layers, which reduces the duplicate frames; (B) RSSD increases the number of feature maps in the multi-scale feature map prediction, increasing the robustness of the detection of small objects.

In the network structure of RSSD, pooling and deconvolution are implemented simultaneously. Before the concatenation, Batch Normalization (BN) operations are performed on the feature maps to unify the feature distribution of the data, thereby improving the detection accuracy.

FSSD emphasizes on the fusion of shallow and deep features. Shallow features have a low semantic level, while deep features have a high semantic level, if they are directly fused, these features will not be able to make full use, and the information loss may occur.

**Figure 9:**
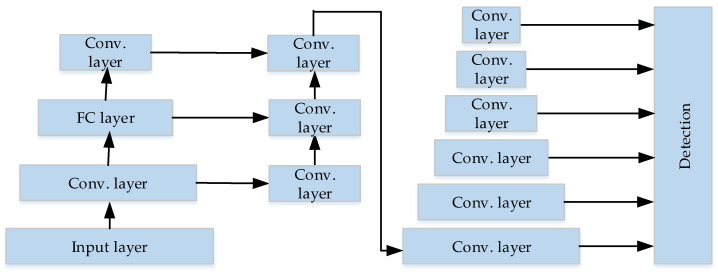
The overall structure illustration of FSSD. The arrows show the information flow. The box of “Detection” is the detector, which output the classes and location of the object in the image or frame.

The basic model of FSSD is basically the same as that of SSD, using VGG-16 as the basic model. The structure of FSSD is shown in Figure 9. The feature map of each layer is resized to the same size to concatenate. In addition, simple block and bottleneck block are used to generate the feature pyramid in the rear stage of FSSD. In the FSSD-512 detection (the input image size is 512 × 512), the detection speed is 35.7 FPS, and the mAP is 84.5%.

### 3.2. Two-Stage Video Object Detection

Since video is composed of frames, theoretically, all two-stage image detection methods could be used for video detection by detecting the frames. In general, since the detection speed of the two-stage detector would be not very fast, this form of video detection cannot implement real-time detection.

Two-stage object detection has a separate module for extracting features and region proposals, which is called backbone. Therefore, the detection speed is slower than the one-stage detector, although the detection accuracy is always higher than the one-stage detector.

**Figure 10:**
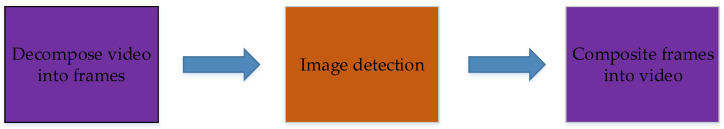
The universal work flow of the two-stage video object detection. The framework is based on the two-stage image object detection, which is the “Image detection” module in the figure.

Now the classic two-stage object detection models include Regions with CNN (R-CNN) [61], Spatial Pyramid Pooling (SPP) Net [62], Fast R-CNN [63], Faster R-CNN [12], ResNet [14], GoogLeNet [45], etc. These models are based on deep convolutional neural networks. The idea of these models is to extract features from images, and classify objects by the trained classifiers. In the stage of extracting features (backbone), the earlier deep learning methods, such as R-CNN, use Selective Search, which slides many boxes in the images, and use neural networks to extract features in these boxes. Selective Search is replaced with Region Proposal Networks (RPN) in Faster-RCNN. RPN generates bounding boxes when extracting features. The idea is applied to other models such as RetinaNet [44]. Since this paper is a review on video detection, please refer to the image detection literatures. The universal work flow of the image-video detection method is shown in Figure 10.

### 3.3. Mixed-Stage Video Object Detection

The mixed-stage object detection is a mixture of one-stage detection and two-stage detection, or other video detections which could not be classified as one-stage or two-stage detection.

**Figure 11:**
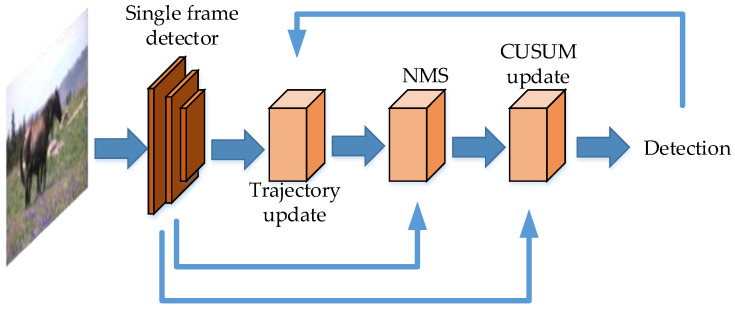
The structure of Minimum Delay video object detection. The single frame detector is a one-stage detector, and the rest is a two-stage detector (include feature extractor and classifier), thus we regard it as a mixed-stage object detector. The structure has two shortcut connections, and a feedback connection, which have improved the detection.

Minimum Delay video object detection [64] uses one-stage and two-stage image detector simultaneously, which can achieve real-time detection speed. The idea of Minimum Delay is the quickest detection theory. The “quickest detection” is to realize fast detection with a probability, by calculating the distribution variation of the video sequences. The “quickest detection” is implemented as the cumulative sum (CUSUM) algorithm. The algorithm of CUSUM integrates the feature map sampling values of the video sequence, and can aggregate the small deviations of the video sequence into a fluctuation. Therefore, CUSUM can detect the changes of the average value of the observed video sequences, and can overcome the signal-to-noise ratio threshold effect. The framework of Minimum Delay Video Object Detection is composed of CNN detector which is implemented frame by frame, an NMS module which is used to filter the inaccurate candidate boxes, the CUSUM module to implement the accurate and minimum delay detection. The CNN detector adopts ResNet [14], SSD, RetinaNet [44], VGG net [58], ZF net [65] in the experiments. The method improves the detection accuracy without reducing the detection speed. When using one-stage detector as the CNN detector, the framework can achieve real-time detection speed. The framework of Minimum Delay video object detection is shown in Figure 11.

**Figure 12:**
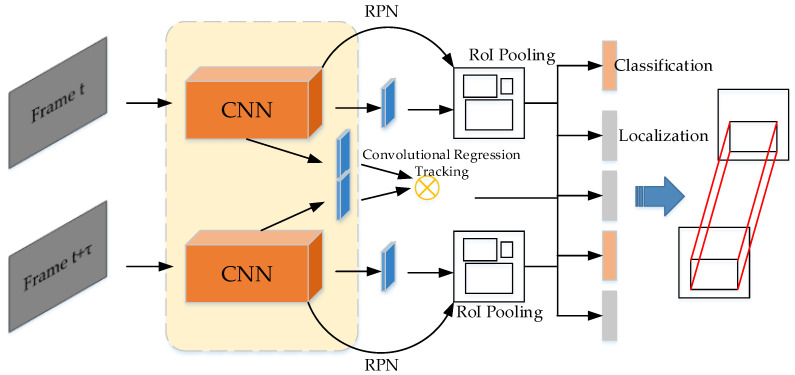
The structure of Convolutional Regression Tracking. Convolutional Regression Tracking is located between the 2 convolutional neural network (CNN) pipelines. The structure can improve the mAP of the image object detector, which can be used as video object detector.

Lyu et al. [66] uses Convolutional Regression Tracking between the adjacent frames for enhancing the video object detector. The structure is shown in Figure 12. Sabater et al. [67] propose a detection refinement method for video object detection, and the refinement method uses a link scoring model to link the feature map of adjacent frames. Bertinetto et al. [68] propose a fully-convolutional Siamese network for the video object tracking, which is light, and outperform the previous object tracking methods. Cai et al. [69] propose Cascade R-CNN, which has a cascade structure, and the cascade structure send the bounding boxes of the previous branch to the next branch. The performance of the method is state-of-the-art. Ustinova et al. [70] propose Histogram loss function for deep embedding learning, and the loss function outperforms the previous loss functions on some important datasets.

Zhang et al. [71] find that, in the multi-object tracking (MOT) system, the detection task and the re-ID task have an interaction, which may affect the re-ID task. They propose a tracking method, which uses an anchor-free single-shot deep network to solve the problem. The network is an unsupervised architecture, and use the input image multiple times for encoding. This makes the network need less training data, and the anchor boxes are not needed in training. Kusetogullari et al. [72] introduces a large-scale handwriting dataset named DIDA, and a deep learning architecture named DIGITNET, which is used to recognize the handwriting in DIDA and other handwriting datasets. DIGITNET is based on YOLO, and followed by three different designed Convolutional Neural Network (CNN) architectures.

Qin et al. apply convolutional neural networks to human behavioral recognition, to constitute an intelligent city system [73]. The proposed approach includes bottom layers, middle layers and top layers, which can locate objects, recognize objects, and recognize the behavior of the objects. The approach has reached good performance in city. Mühling et al. use deep learning method for video content retrieval in films and TV programs, and achieve high retrieval rate in those videos [74]. Hu et al. construct a deep incremental slow feature analysis (D-IncSFA) network, to implement video anomaly detection, which relies on hand-crafted representations [75]. Wang et al. propose a deep learning model to detect video salient regions [76]. They also develop a data augmentation method to simulate the video datasets. Li et al. use deep reinforcement learning method to detect temporal action in videos [77]. They design a long short-term memory (LSTM) structure to generate the features of video sequences. Protasov et al. use a kind of deep convolutional neural network to extract features, then implement semantic video annotation after video scene detection [78].

Wang et al. present three-level hierarchical context modeling, which can recognize the events in videos by using the previous events [79]. Hu et al. propose a deep neural network architecture to enhance person re-identification [80]. The proposed architecture uses neural network to extract the whole and part features of person, and synthesizes the features to realize person recognition. Xu et al. use an unsupervised learning method to detect anomalous events in video surveillance scenes [81]. The method learns person features and their optical flow maps separately after unsupervised encoding and decoding, then uses Support Vector Machine (SVM) [82] to score the events and detect them.

Cao et al. propose a Teacher Network and Student Network architecture, which realize the real-time video detection from vehicle cameras [83]. The Teacher Network is pre-trained, the architecture transfers a layer of the Teacher Network to the Student Network, to make the smaller and simpler Student Network have better performance. Takahashi et al. propose audio event recognition (AER) for video analysis [84]. The audio of the video is used to train a CNN architecture, and then the CNN architecture outputs probability of classes, which will be helpful for video detection, video analysis and subtitle matching. A face video verification system proposed by Chen et al. has four parts: face detection, face association, face alignment, face verification [85]. The method synthesizes the previous face detection methods, and the detection effect is better.

Zheng et al. propose a video dynamics detection method that can detect the events in videos [86]. The method combines Deep Neural Network (DNN) and Recurrent Neural Network (RNN), increases the detection accuracy, and reduces the training time. A novel convolutional neural network architecture is proposed to detect the event of action in videos [87]. The architecture is called Tube Convolutional Neural Network (also abbreviated as T-CNN), which uses 3D convolution on videos, extends 2D convolution and pooling to 3D, to generate 3D video Region of Interests (RoIs). Yao et al. propose a kind of deep learning method to detect object-based forgery [88]. The method detects video frames by using convolutional neural network (CNN) after a predefined high pass filter, and achieves better performance than in existing literature.

Wang et al. integrate the method of object detection into video saliency detection, propose a feature hybrid framework to detect the spatiotemporal saliency in videos [89]. Their method also works in video frames. Niu et al. propose an architecture to detect fake face or masked face from normal face video frames, which first aligns the faces and extract facial features, then utilizes Gaussian Mixture Model (GMM) to classify faces [90]. GMM is a model that implements clustering through Gaussian probability function. The method achieves real-time speed and high accuracy. A deep learning-based detection method called NB-CNN detects video frames of reactors’ cracks [91]. NB-CNN uses convolutional neural network and Naive Bayes classifier. This method has reached a good level among similar algorithms.

Li et al. consider the background and foreground of the video frames both, and implement background extraction and foreground detection [92]. The approach is named Hierarchical Modeling and Alternating Optimization (HMAO). Tao et al. [93] uses signal processing methods to detect smoky vehicles on the road, such as Grey-Level Co-Occurrence Matrix (GLCM) [94], Discrete Wavelet Transform (DWT) [95]. The detection speed is high, and the method can use the videos taken by the car camera to detect. Bilal et al. [96] propose a method to detect pedestrian in an efficient way, the method is made up of cascaded Support Vector Machine (SVM). The detection accuracy and speed are better than other similar methods.

A novel convolutional neural network (CNN) architecture is proposed to recognize human action in videos [97]. The architecture first extract action region of video frames, and then use CNN combined with optical flow algorithm. The Siamese region proposal network (Siamese-RPN) is proposed to track the objects in videos by Li et al. [98]. The videos are converted into frames, then the frames are divided into template frames and detection frames. Siamese-RPN is a parallel connection of the template frames and detection frames. The tracking speed is about 160 FPS.

Diba et al. present a kind of 3D convolution to extract the features of videos [99]. It performs convolution operations on multiple video frames simultaneously. The method is more expressive and efficient for multiple video frames. Nascimento et al. propose a framework which can evaluate the detection effect via frames of videos [100]. This framework is applied to the detected image frames through a series of templates.

Zhou et al. present a novel unsupervised learning architecture, to automatically learn the features of Depth and Ego-Motion from videos [101]. The architecture is made up of a kind of Depth CNN and Pose CNN, the target frame is learned through Depth CNN, the previous frame, the next frame and the target frame pass to the target frame through CNN. The unsupervised learning method is comparable to those supervised learning methods. Feichtenhofer et al. combine two Residual Networks together with a kind of multiplicative interaction, to perform the spatiotemporal video action recognition [102]. The method has a state-of-the-art detection effect on dataset UCF101 [17] and HMDB51 [31].

Liu et al. propose an unsupervised learning architecture named Deep Voxel Flow (DVF) [103]. DVF uses an encoder network and a decoder network to generate the voxel flow of a video. The approach achieves state-of-the-art in video frame synthesis. Zhu et al. present a novel detection method of person re-identification in videos [104]. The method is named Simultaneous Intra-video and Inter-video Distance Learning (SI^2^DL), which uses intra-video distance metric and inter-video distance metric to learn the features. Intra-video distance metric is used for learning the features within a video, and inter-video distance metric is used for learning the features between videos. The method is also implemented on the video frames. An unsupervised learning representation called DRNET is proposed by Denton et al., used for many tasks, such as predictions of future frames in videos [105]. The model uses a content encoder and a pose encoder to learn the content feature and the human pose feature, and uses a novel adversarial loss, which is similar to Generative Adversarial Networks (GAN) [106].

Fast YOLO [107] adopts probabilistic genetic encoding modeling strategy, and motion-adaptive inference, and the architecture can be used in embedded systems. Galteri et al. apply a closed-loop structure to object detection such as EdgeBoxes [108], BING [109], RPN [12], which has achieved a superior detection effect [110]. The key of the closed-loop structure is the feedback function, which feeds back the detected information to image by multiplying IoU and the detection score. Wang et al. analyze the process of convolution and find that lower layers of convolution have more details of object features, higher layers of convolution have high semantic features [111]. They also find that most of the feature maps are irrelevant to the detected objects. These findings provide useful insights for video detection.

Yuan et al. proposed a framework which can efficiently detect the traffic sign in videos [112]. The method uses the video frames as the input, first, the traffic signs are located through Aggregated Channel Features (ACF) detection, which can aggregate the features from different convolutional channels. Second, the state of the traffic signs is estimated by a proposed function, to make some modifications, and then the deposed signs are detected by an online detector with KF model, if they are not detected in the first step. Deng et al. propose an external memory method, when the detection system needs to store long term temporal information [113].

Chen et al. present a set of video detection metrics, to assess the dynamic detection effect of videos, such as center jitter error (CJE) and size jitter error (SJE) of them [114]. Bengar et al. propose a kind of active learning method, which is used to assist in annotating video data [115]. The method detects the un-annotated video frames, and uses adjacent frames to locate the object in the current frame. Yang et al. add Temporal Context Module and Spatial Context Module into the multiple image object detectors, for the usage of detecting wild great apes [116]. Literature about the UG^2^+ (UAV, Glider, Ground) challenge concludes that the methods with better video detection effect use spatiotemporal context method [117].

Luo et al. use spatial-temporal context aggregation (STCA) to fuse the feature maps [118]. STCA learns the spatial-temporal information from the object proposals both within a frame and among the adjacent frames. Shankar et al. study the impact of the video perturbation to the detection accuracy, and find that the previous frame would have a negative impact on the next frame, resulting in a detection error of the next frame [119]. Wang et al. design a statistical convolutional neural network (SCNN), and the convolutional neural network is composed of coefficient vectors and deterministic weights [120]. Chin et al. find that the lower resolution image can produce better accuracy sometimes, and they propose AdaScale to reduce the frame resolution, and select the smallest loss frame scale to train and detect [121]. Kumar et al. invented an algorithm that can integrate the Regions of Interest (RoIs) of the adjacent frames to one, to detect the object in the RoI [122]. The algorithm can reduce the computational FLOPS.

## 4. Extracting the Key Frames for the Video Detection

Han et al. invented Seq-NMS to find the most accurate region proposals from a video clip [123]. The method selects the highest score of a frame sequence from a video, rescores the sequence, and removes the overlapped region proposals. Seq-NMS placed high rank in the video object detection (VID) task of the 2015 ImageNet Large Scale Visual Recognition Challenge.

Scale-Time Lattice (ST-Lattice) [124] also does not implement convolution, feature extraction and detection on non-key frames, and does not use optical flow. It propagates the key frame to the non-key frames via Motion History Image (MHI). This method uses the idea of recursion, starting from the key frames of the beginning and the end, detects the non-key frame in the middle of two key frames via MHI, and then divides them into two segments by the non-key frame, and detects the residual non-key frames in the middle of these two segments, and then divides them into four segments, recursively detects them until all the non-key frames are detected.

Pouyanfar et al. propose a kind of deep learning architecture to detect the semantic event of videos [125]. They extract the features of a video frame and detect it via Support Vector Machine (SVM), after the key frame selection. Luo et al. invented a scheduler network to select which frame is the key frame, and the network detects the key frames and tracks the non-key frames [126].

## 5. Video Detection Using the Temporal Information

The following methods use the temporal information by Long Short-Term Memory (LSTM) [16], by optical flow [15], or use convolution on multiple frames in chronological order, such as 3D convolution [127]. Some use traditional video detection methods such as optical flow on the adjacent frames, such as FGFA [128]. Some implement image detection methods such as R-CNN on the neighbor frames, such as T-CNN [129].

### 5.1. LSTM-Based Video Detection

#### 5.1.1. Association Long Short-Term Memory (Association LSTM)

Long short-term memory (LSTM) [16] is suitable for learning the features with temporal information, because of the connectivity of the structure. LSTM processes multiple video frames simultaneously, and also has the memory characteristics. Association LSTM [130] is proposed by Lu et al. in 2017, which could be considered as the combination of SSD and LSTM.

**Figure 13:**
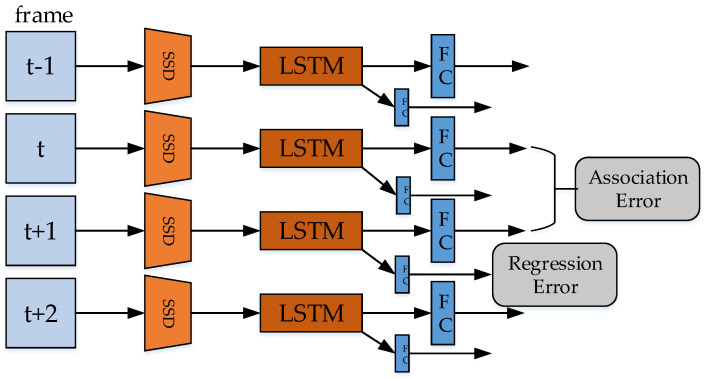
The architecture of Association LSTM. SSD is a one-stage detector, which is described before. FC is the fully connected layers. Association Error generates the object classification, Regression Error generates the object localization.

The architecture of Association LSTM is shown in Figure 13. The video frames are first sent to SSD network to extract the features, then stacked and sent to LSTM for training. Association LSTM uses an additional association error loss function. Since SSD can extract features quickly, the consumption of association LSTM in training may not increase much.

#### 5.1.2. Temporal Dynamic Graph LSTM (TD-Graph LSTM)

TD-Graph LSTM [131] is a weakly-supervised video object detection framework. The framework adds a Temporal Dynamic Graph Construction before Long Short-Term Memory (LSTM), to enhance the temporal information between the original video frames. The proposed approach has an advantage on object motion recognition, i.e., the recognition of the label of human actions.

**Figure 14:**
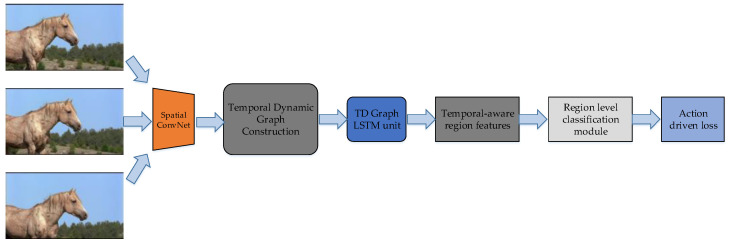
The workflow of TD-Graph LSTM.

The workflow of TD-Graph LSTM is shown in Figure 14. The adjacent frames are sent into Temporal Dynamic Graph Construction via Spatial ConvNet, a kind of CNN which is derived from Fast RCNN. Then the generated feature maps are sent into TD-Graph LSTM unit and Region-level Classification Module. The method has a good performance on Charades dataset [37].

#### 5.1.3. Bottleneck-LSTM

Bottleneck-LSTM [132] is a lightweight LSTM, a video object detector running with 15 FPS on the mobile terminal, and detects every frame. This lightweight structure can speed up the propagation, and the refinement of features between the video frames.

Bottleneck-LSTM is a combination of LSTM and SSD, i.e., SSD is integrated into the front and back of LSTM, and the gates of LSTM, such as the forget gate, the input gate and the output gate, are improved to make it more efficient, and more suitable for mobile terminal.

#### 5.1.4. Patchwork

Patchwork [133] introduces recurrent attention models into video object detection. Similar to LSTM, patchwork transfers the information from the previous frame to the next frame, adds attention mechanism into the network, and the attention is transmitted to the next frame. For each frame detection, patchwork uses the attention module of the previous frame to extract the sub-window of each frame, and only detects this sub-window.

Since each frame only detects a part of the frame (which is the sub-window), in order not to omit some important information, patchwork combines the feature map of the previous frame with the feature map of the sub-window by the patchwork cell. In this way, detection of sub-window improves the detection speed, while patchwork cell makes the network not omit the entire frame information. Patchwork also uses a Q-learning method [134] to enhance the location of the object. Furthermore, patchwork has a low detection latency compared with other video detection methods.

#### 5.1.5. Progressive Sparse Local Attention (PSLA)

Guo et al. propose the Progressive Sparse Local Attention (PSLA) [135] to transfer the feature maps between frames. This method achieves good detection results on ImageNet VID dataset, with a smaller size model.

The detection structure of PSLA is similar to LSTM. Different from LSTM, the recursive module of this structure uses the proposed Recursive Feature Updating (RFU) and Dense Feature Transforming (DenseFT). The core of RFU and DenseFT is the proposed PSLA. Both RFU and DenseFT are used to transfer temporal information from the previous frame feature map to the next, RFU is used on key frames, while DenseFT is used on non-key frames.

Two kinds of mapping are used in the implementation of PSLA. One is to use a sparse matrix to scatter each feature map cell to the periphery. The advantage of this is to enhance the neighbor information of each cell. The other is to integrate the results of the first step with the corresponding weights. The approach gets 81.4% mAP on ImageNet VID.

#### 5.1.6. Mobile High Performance Video Object Detection

Zhu et al. present a light weight video object detection architecture with high performance [136]. The architecture is also similar to LSTM [16], and the difference from LSTM is that the proposed architecture adds Light Flow network between the columns of LSTM. The Light Flow network is derived from FlowNet [137], which transfers feature maps by optical flow.

The detection network of the proposed architecture adopts RPN [12] and R-CNN [61], and the feature network adopts MobileNet [138], however, the last pooling and the fully-connected layer of MobileNet are removed. The system achieves 60.2% mAP score on ImageNet VID validation dataset, with 25.6 FPS of Huawei Mate 8.

#### 5.1.7. Learnable Spatio-Temporal Sampling (LSTS)

The optical flow method is often used to transfer feature maps. Jiang et al. proposed Learnable Spatio-Temporal Sampling (LSTS) [139]. Different from the optical flow method, LSTS integrates the feature map into other frames by a certain weight, and this weight can be learned through iterations in the model training.

The framework of the proposed system is also like LSTM. The key part of the system is Sparsely Recursive Feature Updating (SRFU) and Dense Feature Aggregation (DFA), SRFU is used for key frames, and DFA is used for non-key frames. The LSTS module is integrated into SRFU and DFA. The proposed system has good detection results on ImageNet VID dataset, with 82.1% mAP score and less computation time.

#### 5.1.8. LiDAR-Based Online 3D Video Object Detection

The proposed approach [140] is applied to point cloud videos. Point cloud video is generated by Light Detection and Ranging (LiDAR), which contains 3D coordinates X, Y, Z, color, time and other information. The method adopts the idea of LSTM, replacing the key nodes of LSTM with Attentive Spatiotemporal Transformer GRU (AST-GRU), a proposed structure embedded with Spatial Transformer Attention (STA) module and a Temporal Transformer Attention (TTA) module.

The AST-GRU module is also used for extract the spatiotemporal relationship between the adjacent frames. The STA in AST-GRU is used to detect the foreground objects, and the TTA in AST-GRU is used to detect the dynamic objects.

The proposed system has a good performance on nuScenes dataset [141], which is a large-scale automatic driving dataset, not only contains the data of camera and LiDAR, but also records the radar data.

#### 5.1.9. Memory-Guided Mobile Video Object Detection

Liu et al. develop a temporal memory fusion module from LSTM, to fuse the feature maps of adjacent frames [142]. The feature extraction network adopts interleaving slow network and fast network. This method uses reinforcement learning to establish an Adaptive Interleaving Policy Network to determine which feature extraction network (the slow network or the fast network) to run.

The approach achieves good performance on the dataset of ImageNet VID 2015, compared with other mobile video detection methods. The running speed of the proposed system can reach more than 70 FPS on PIXEL 3.

#### 5.1.10. Two-Path Convolutional LSTM (convLSTM) Pyramid

**Figure 15:**
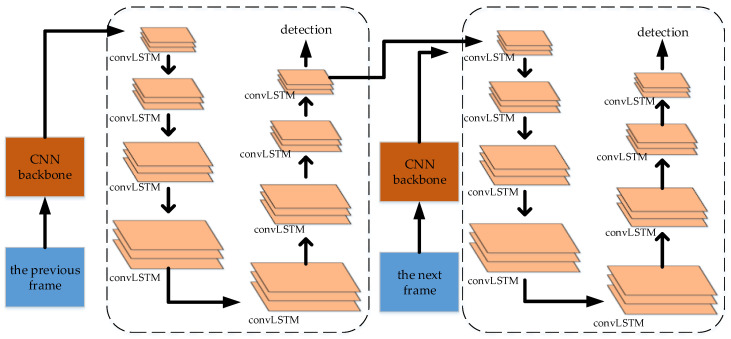
The structure of Two-Path convLSTM Pyramid. The detection result of the previous frame is aggregated into the detection process of the next frame.

Zhang et al. [143] proposed a pyramid LSTM structure for the video frame detection. This structure combines LSTM into a pyramid structure. The workflow is that a CNN backbone is used to extract the features of a frame, and then the features are sent to the LSTM pyramid structure for detection. The previous frame and the next frame are detected respectively with two LSTM pyramid structures, and the two paths of the LSTM pyramid structures are connected. This structure has the state-of-the-art result in the ImageNet VID dataset. The structure is shown in Figure 15.

#### 5.1.11. Spatial-Temporal Memory Network (STMN)

**Figure 16:**
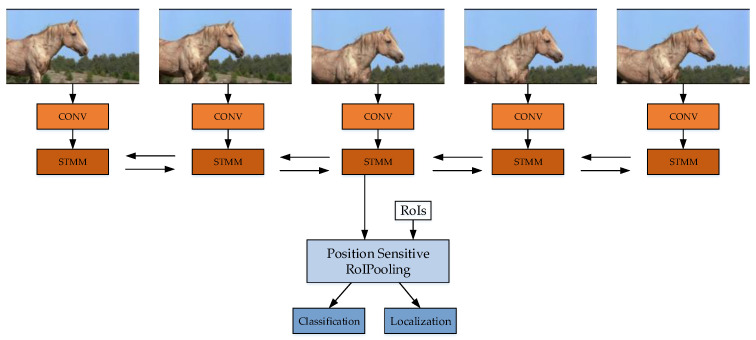
The structure of STMN. Spatial-Temporal Memory Module (STMM) can extract and transmit the spatial-temporal features. STMN does not use the fully connected layer after Position Sensitive RoI Pooling.

Spatial temporal memory network (STMN) [144] may be a kind of Recurrent Convolution Network (RNN) [145]. STMN convolutes the video frames at the beginning, and obtains the spatial features, then sends them to Spatial-Temporal Memory Module (STMM), and then the classification and regression network via position sensitive pooling. Since different STMMs are connected, the temporal features of the feature map are obtained via STMM. Since STMM is a bidirectional circular neural network, it may be able to learn the motion information in a longer period of time. The structure of STMN is shown in Figure 16.

A Tubelet Proposal Network (TPN) [146] is presented by Kang et al., to generate long tubelet proposals more efficiently. The tubelet is a cubic tube formed by the same object in adjacent frames. A Long Short-term Memory (LSTM) network is adopted to combine the tubelet proposals, and the detection accuracy is high.

### 5.2. Video Detection Using Optical Flow

#### 5.2.1. Tubelets with Convolutional Neural Networks (T-CNN)

T-CNN [129] is proposed by Kang et al. T-CNN is composed of tubelet extraction module, tubelet classification module and tubelet re-scoring module. One of the innovations of T-CNN is the tubelet in video convolution. Tubelet is formed by combining the bounding boxes of the same object in adjacent frames. The construction of tubelet consists of three steps. Firstly, the image object detection method is used on the video frames, and the bounding boxes are extracted by Selective Search; secondly, the bounding boxes are scored and classified by R-CNN; thirdly, the bounding boxes with high confidence scores are combined to form the tubelet.

**Figure 17:**
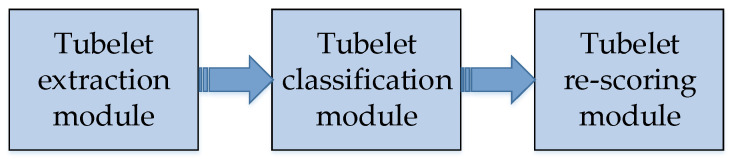
The flow chart of T-CNN.

T-CNN uses optical flow to adjacent video frames. T-CNN calculates the average optical flow vectors in the region proposal boxes. This may improve the robustness of video detection. The flow chart of constructing tubelet is shown in Figure 17. The three steps of constructing tubelets in T-CNN is illustrated in the figure. While scoring the bounding box, the average weighted optical flow method is used to strengthen the detected frame. Finally, the generated tubelet is filtered by Non-Maximum Suppression to leave the highest score tubelet.

T-CNN achieved good detection results on ImageNet VID dataset and YouTube Objects (YTO) dataset, and won the VID challenge in the ImageNet Large-Scale Visual Recognition Challenge (ILSVRC) 2015.

#### 5.2.2. Deep Feature Flow (DFF)

This paper [147] combines deep learning with optical flow. Deep Feature Flow only detects the key video frames. For the non-key frames, the feature map is transmitted from the feature map of key frames by optical flow. Then, the transmitted feature map is detected by task net (Net_task_). Since only the key frames are convoluted, DFF reduces the computation of video detection.

**Figure 18:**
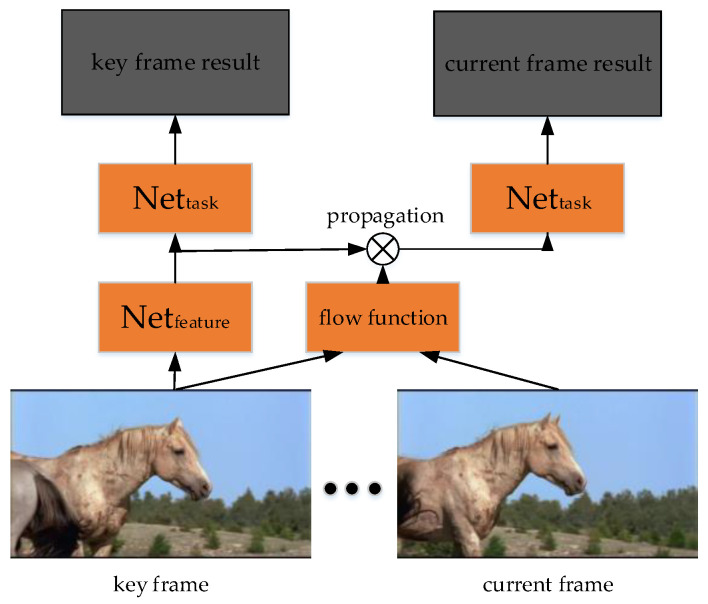
The illustration of DFF. Net_feature_ is a feature extractor (backbone), Net_task_ is a detector, flow function is the optical flow net [137]. The video frames are from YTO dataset.

ResNet-50 or ResNet-101 [14] is used as the feature extraction network, R-FCN [148] is used as the recognition network, and the simple version of FlowNet [137] is used as the optical flow network. Since the computation of FlowNet is much faster than the convolutional network, it can improve the speed of non-key frame detection. The structure of DFF is shown in Figure 18.

Another study by Zhu et al. [149] uses key frame adjustment, which is different from DFF using fixed key-frames. The method of adjusting the key frame avoids selecting frames that are too blurry, or have too little difference from the previous key frame.

Key frame adjustment generates the feature consistency metric to measure whether the two frames have obvious optical flow motion. If the offset is too large, this frame will be taken as a key frame, and the feature will be extracted by image object detection method; if the offset is less than the threshold (0.2 in the literature [147]), it means that the frame is measured as a non-key frame, a DDF method will be used to calculate the feature map and detection result of the non-key frame.

#### 5.2.3. Flow-Guided Feature Aggregation (FGFA)

FGFA [128] finds that the detection accuracy of blurry frames can be improved by aggregating the feature maps of adjacent frames. The method is proposed by Zhu et al. in 2017. FGFA uses optical flow to aggregate the feature maps.

FGFA includes the modules of feature map extraction, optical flow transmission and feature map aggregation. The feature map extraction module is called backbone in some literatures, which is ResNet-50, ResNet-101 [14] and Inception-ResNet [150] in the paper. The optical flow module uses FlowNet [137], which transfers the feature map of adjacent frames. The feature map aggregation module uses the weighted sum algorithm. In the paper, the cosine similarity algorithm is used to describe the similarity among the feature maps, and used as the weight in the weighted sum algorithm.

**Figure 19:**
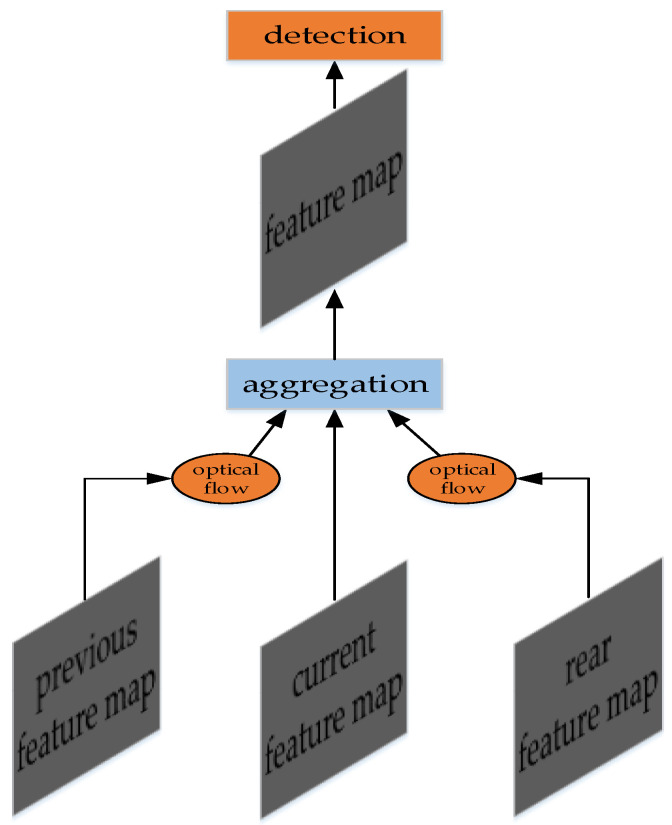
The structure of FGFA. Optical flow is a feature transmission method.

The structure of FGFA is shown in Figure 19. Although good results have been achieved in the detection, we consider that FGFA is of great significance for blurry video detection.

#### 5.2.4. Fully Motion-Aware Net (MANet)

MANet [151] is proposed by Wang et al. in 2018, which is also a method to fuse different feature maps by optical flow. The discovery of this paper is that the combination of global features, local features by optical flow can improve the video detection. MANet uses optical flow to fuse the global features and local features on adjacent frames, and fuses the feature maps of global features and local features. After that, the feature maps are trained and tested. MANet has a good performance in the experiment.

#### 5.2.5. Long Short-Term Feature Aggregation (LSFA)

Long Short-Term Feature Aggregation (LSFA) [152] is proposed by Wang et al. in 2021. This method may refer to the idea of FGFA. LSFA has two parts, which perform detection separately: one part is a long-term feature aggregation, the other part is a short-term feature aggregation. Long-term feature aggregation uses a large feature extraction network, and short-term feature aggregation uses a tiny feature extraction network.

In long-term feature aggregation, key frames and non-key frames are fused by optical flow method. After the key frame goes through the large feature extraction network, a feature map is generated. The generated feature map is then fused with the previous fused feature map, and finally sent to R-FCN for detection.

**Figure 20:**
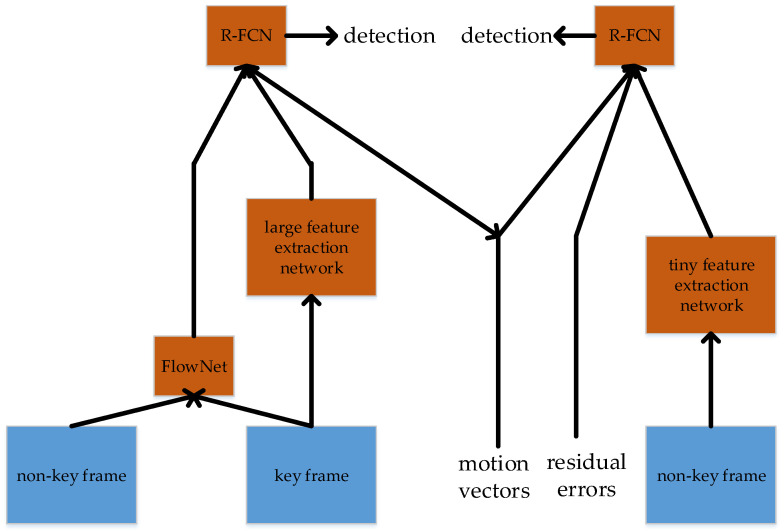
The structure of Long Short-Term Feature Aggregation (LSFA). Flow Net implements the optical flow method, large feature extraction network extracts the complex features from the key frame, tiny feature extraction network extracts the simple features from the non-key frame.

In short-term feature aggregation, motion vectors and residual errors are calculated from the non-key frames and key frames. After that, motion vectors, residual errors, and the feature maps generated in long-term feature aggregation are fused with the non-key frames, and then R-FCN is used for detection. This method has achieved good results on the large-scale ImageNet VID benchmark, and meanwhile improved the detection speed. The structure is shown in Figure 20.

The literature [153] is to convolute the original frames and the optical flow of the frames respectively, go through the pooling layer and LSTM, respectively, and finally fuse the two results to get the classification results. Another method is to fuse the frames and the optical flows after going through CNN separately [154]. The improved version is that the frames and the optical flows goes through CNN, then goes through LSTM, and finally fused to classify [155]. The above methods take advantage of the optical flow, which may be one of the directions of video object detection.

### 5.3. Video Detection Using Convolution among Adjacent Frames

Convolution among adjacent frames is generally used for video object tracking. The idea of video tracking is generally convolution among adjacent frames first, and aggregation second. Some methods are to convolve adjacent frames sequentially for extracting the temporal features, such as 3D convolution [127].

#### 5.3.1. 3D Convolution

Three-dimensional convolution [127] was proposed by Ji et al. in 2012, which is used to recognize the motion state of objects. Three-dimensional convolution integrates the spatiotemporal feature information of frames. The convolution architecture can generate multi-channel information from adjacent video frames, and perform convolution and down-sampling operations on each channel separately, and finally combine the information of all channels to obtain the final feature description.

**Figure 21:**
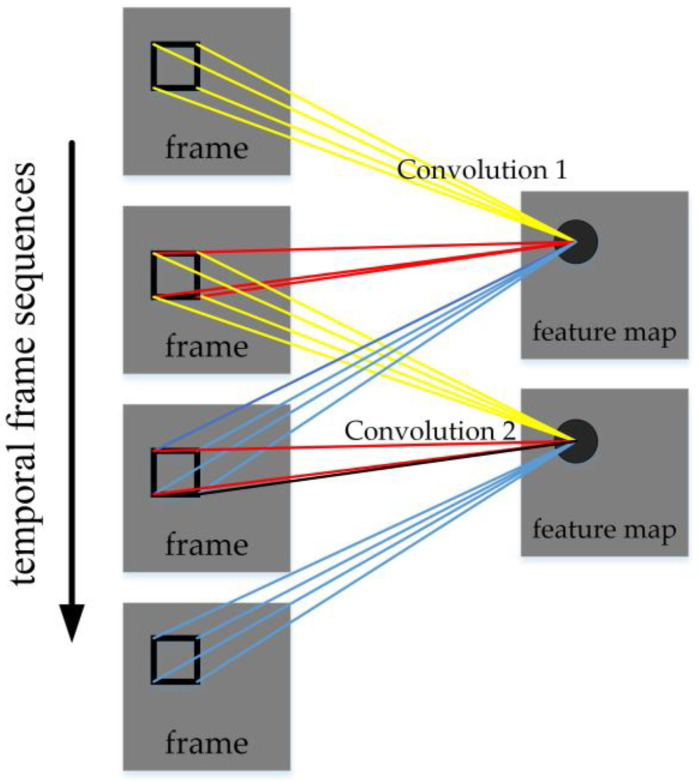
Three-dimensional Convolution. Every 3 adjacent feature maps are convolved to the next feature map, and move on in this style.

The ways of extracting temporal information include: using LSTM to operate multiple adjacent frames [130,131], combining the same object of adjacent frames [129], etc. Three-dimensional convolution [127] is to make convolution on every three adjacent frames, and move the stride of one frame in chronological order to do the convolution repeatedly. After that, the process is similar to the conventional convolution, and finally the full connection layer. The advantage of 3D convolution is that it might fully extract the small differences of objects in different frames. For high-precision video detection, using this method to train the model might be effective. Figure 21 illustrates 3D convolution.

#### 5.3.2. Temporal Convolutional Network (TCN)

Temporal Convolutional Network (TCN) [156] is proposed by Bai et al. in 2018. The authors prove that convolutional neural network can be used for modeling with temporal information, compared with Recurrent Neural Network (RNN) [145]. RNN is usually used for modeling with temporal information, because the cyclic structure of RNN is suitable for representing temporal information.

**Figure 22:**
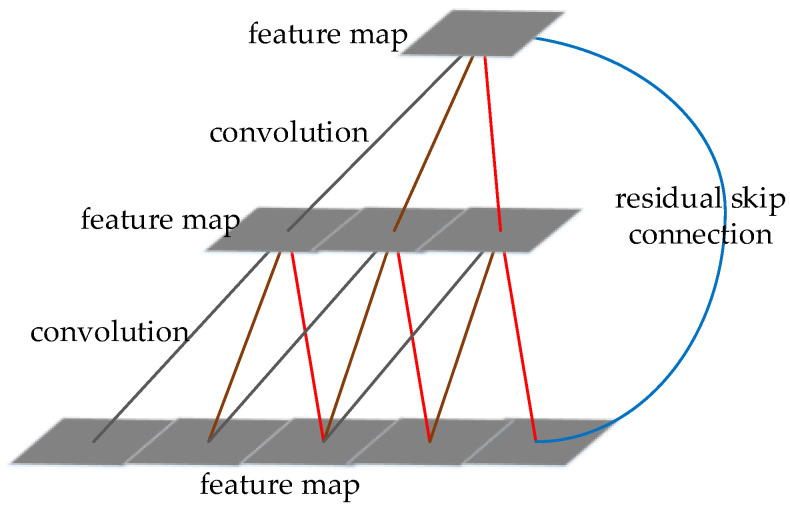
The structure of TCN. Every 3 adjacent feature maps are convolved to the next feature map. The blue connection is a residual skip connection.

TCN merges and convolves adjacent frames, and then convolutions after sampling. In addition, the shortcut in ResNet is added to the convolution. The structure of TCN is shown in Figure 22. In the experiment, TCN performs better than the original LSTM [16], GRU [157] and RNN [145] in multiple tasks, it takes less memory and converges better than RNN.

#### 5.3.3. Detect to Tracks and Tracks to Detect (D&T)

**Figure 23:**
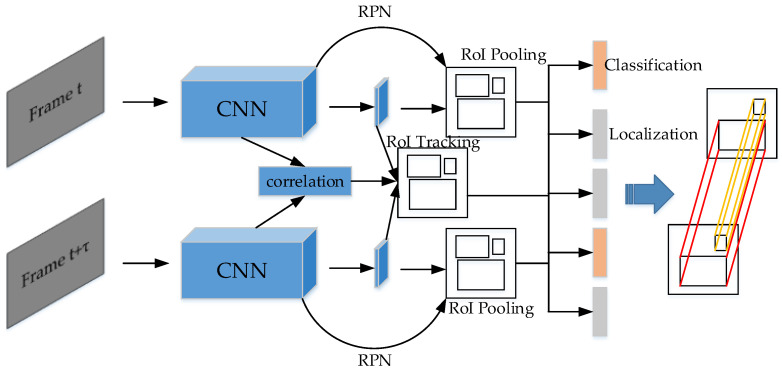
The illustration of Detect to Tracks and Tracks to Detect. The two CNN pipelines are correlated for the RoI Tracking, for the purpose of enhancing the video object detection.

The idea of this paper [158] is that two frames of a certain time interval are convoluted and extracted the features by the backbones, then sent into two branches. One is to use the structure of Faster R-CNN [12] to classify the objects and regress bounding box, and the other is to use the correlation network to calculate the correlation features. Next, the correlation features are combined with the features of the above Faster R-CNN features to generate RoI tracking. The final output also contains two outputs: one is the class and bounding box of the objects in the frames, and the other is the trajectory of each object in different frames generated by RoI tracking. The illustration is in Figure 23.

#### 5.3.4. Recurrent Residual Module (RRM)

RRM [159] uses the relationship between adjacent frames to speed up the calculation of CNN, thereby improving the detection accuracy and speeding up the detection. The idea of RRM is to subtract the repeated elements of adjacent frames, and only detect the changed information between video frames. This may be conducive to the detection of moving objects, because the background may be often the repeated.

**Figure 24:**
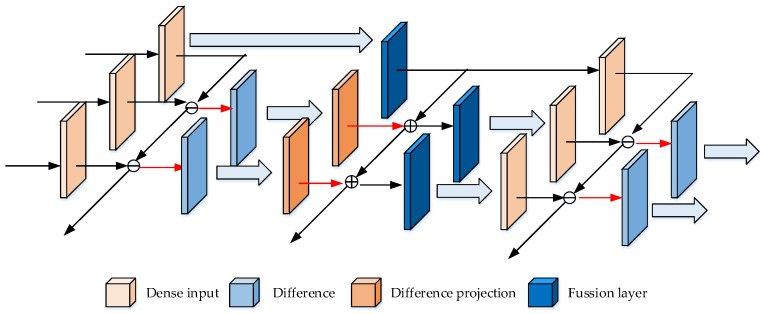
The structure of RRM. ⊖ represents the feature map subtraction operation, which can highlight the differences among the adjacent frames. ⊕ represents the plus operation, which can highlight the similarities among the adjacent frames.

The illustration of RRM is shown in Figure 24, the adjacent frames are subtracted to the second layer, and the feature maps of the second layer after convolution are added to the next layer, and repeat. The operation of addition may be regarded as a kind of fusion, and the effect may be to combine the features of the background (which is kept in the tensor without subtraction, in the first line of Figure 24) and the foreground (which is subtracted to extract in the previous layer), to make the detection result more accurate.

#### 5.3.5. Spatiotemporal Sampling Network (STSN)

STSN [160] extracts features from the reference frame and the supporting frame, and goes through multiple deformable convolutional modules. Then, STSN classifies the objects after the deformable convolutional layers.

Since STSN combines the reference frame and the supporting frame for detection, this may have a significant detection effect on the video part occlusion and motion blur, for the reason that there are always clear objects in the frames.

#### 5.3.6. Integrated Video Object Detection and Tracking

Integrated Video Detection and Tracking [161] uses the idea of multi-object tracking. A bounding box pool is used to store the results of the previous frames. These results are associated with the current frame, to contribute to the classification and scoring of the current frame.

The framework of this method adopts the structure which is similar to Faster R-CNN [12]. The structure detects the current frame, and adds the bounding box information of the previous frame into the current frame, to form the track, and then outputs the bounding box to the next frame. This method achieves 83.5% detection mAP and 72.6% tracking mAP on ImageNet VID validation set.

#### 5.3.7. Relation Distillation Networks (RDN)

RDN [162] uses a detector (similar to Faster RCNN) to generate RoIs from multiple video frames, and uses the relation module to combine the generated RoIs, finally the feature maps are sent to a detection network for classification and location regression.

The relation module is derived from the literature [163]. Objects are closely related to the surrounding environment, and the combination of surrounding information often has a favorable impact on object detection. The relation module is used to combine the object with the surrounding neighborhood information. “Neighborhood” refers to the adjacent background information of the object. The relationship module concatenates the information of the object and its neighboring background with the coordinate information of the object, and fuses with the object features detected before to obtain the final object features. RDN fuses the top proposals of an object, and wins 84.7% mAP on the dataset of ImageNet VID.

#### 5.3.8. Long-Range Temporal Relationships

For video detection, motion blur, occlusion, and object deformation are often the difficulties. The temporal information is often one of the effective methods to solve these difficulties. The architecture is proposed by Shvets et al. It uses the relation block between frames to learn the correlation information among frames, which improves the accuracy of video object detection, and the detection consumption does not increase much [164].

The structure of the relation block is described as follows. The target feature map and the support feature map are embedded in the linear layer. After the normalization, the matrix multiplication, the softmax layer and linear conversion layer, the both feature maps are concatenated to obtain the synthesized relation block feature map. The framework reaches good detection accuracy on ImageNet VID dataset, reaching 84.1% frame mAP value.

#### 5.3.9. Sequence Level Semantics Aggregation (SELSA)

The difficulties of the video detection are motion blur, part occlusion, and object deformation, etc. One of the ideas which could solve the problems is to transfer the feature maps from clearer frames to the blurry frames. Traditional methods often use optical flow or Recurrent Neural Network (RNN) to transfer the feature maps. Wu et al. proposed the novel Sequence Level Semantics Aggregation (SELSA) [165] for feature map transfer. The advantage of this method is that the feature map transfer can be performed on all the video sequences, not only the adjacent frames.

SELSA uses a novel algorithm to calculate the similarity of the region proposal box between frames, calculates the weight from the similarity by another proposed algorithm, and integrates the feature map of the support frame into the current frame by the calculated weight.

The framework uses Faster R-CNN [12] to extract the proposal boxes and detects them, and the SELSA module is added after the fully-connected layer of Faster R-CNN. The video frames are processed by the framework, and are fused to the middle tested frame to detect. The detect effect is superior compared with the test algorithms in the literature [165]. The proposed system has a good detection accuracy on the ImageNet VID and the EPIC KITCHENS dataset [166], and the architecture of the system is simpler compared with other video detection systems.

#### 5.3.10. Detection System for Extended Video Analysis

**Figure 25:**
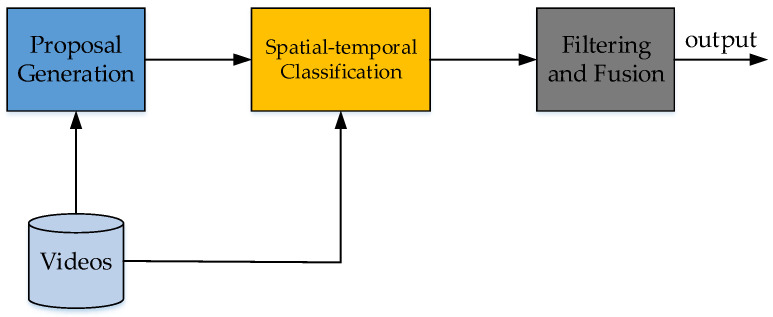
The workflow of proposed video detection system.

Liu et al. propose a video detection system [167] to execute the video analysis in the video surveillance application. The system has three stages: proposal generation, spatiotemporal classification and post process, as shown in Figure 25. The video detection should be based on the frame by frame method. Except for the conventional image detection method (extracting region proposal boxes from an image, extracting features in the boxes and detecting), a video scene judgment module and an object activity feature fusion module from different frames are added. The method wins TRECVID Activities in Extended Video (ActEV) challenge 2019.

#### 5.3.11. Memory Enhanced Global-Local Aggregation (MEGA)

MEGA [168] combines the global semantic information of adjacent frames with the local information of a single frame, such as the location, shape and size of the object in a frame. Previous methods either focus on the global semantic information or the local information of a single frame. Additionally, MEGA measures the two aspects synthetically to execute video object detection.

**Figure 26:**
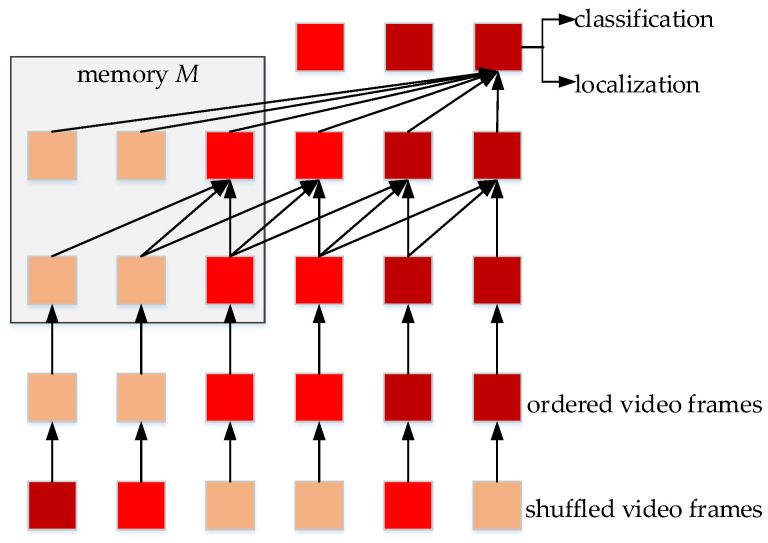
The illustration of MEGA. The arrows denote the directions of the aggregation. The depth of the color indicates the sequence.

The architecture of MEGA is illustrated in Figure 26. The ordered video sequences and the shuffled sequences are aggregated together to the next part. The next part of MEGA is to aggregate the three adjacent feature maps of the first step, the number 3 is the memory size. Next, the aggregated feature maps are integrated to the key frames, and the object classification and location regression are implemented. MEGA has a good video object detection on the ImageNet VID dataset using the backbone of ResNeXt101 [169].

#### 5.3.12. Temporal Shift Module (TSM)

**Figure 27:**
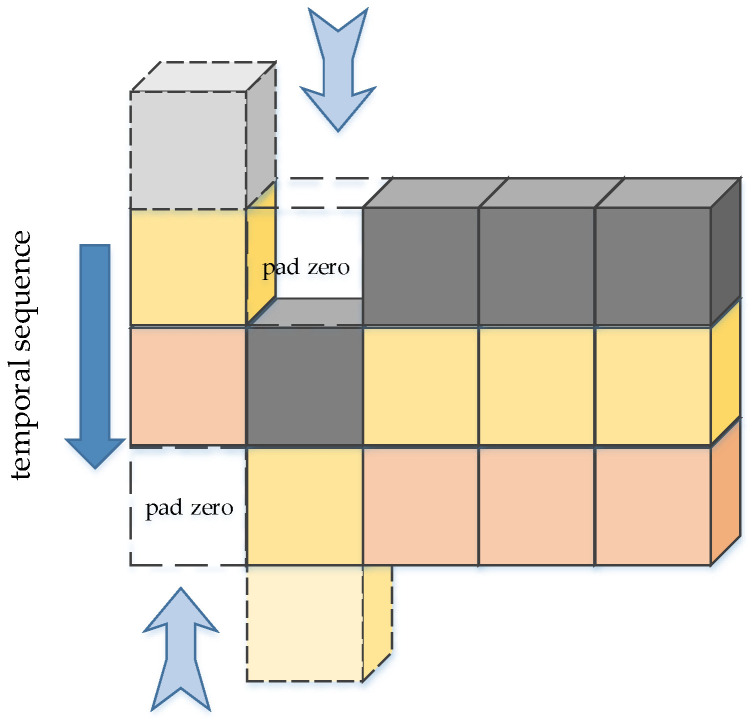
The illumination of TSM. TSM shifts the feature map tensor along the temporal sequence, forward or backward. The empty positions are filled with zeros.

Lin et al. [170] develop a method that consumes less resources, and can realize temporal module of adjacent video frames, Temporal Shift Module (TSM). TSM shifts the feature map tensor along the temporal dimension. We regard this shift as a kind of shuffle before convolution among adjacent frames. Since the shift operation may not spend much computing power, TSM can run fast for video object detection. TSM has a good video detection performance on the datasets of Kinetics [38], UCF101 [17], HMDB51 [31], Something-Something [42] and Jester [171]. The illustration of TSM is shown in Figure 27.

#### 5.3.13. Context Region with Convolutional Neural Network (Context R-CNN)

For the videos with a long-time span, such as the hidden video camera for filming wild animals, and the video surveillance of the traffic conditions, their backgrounds are always the same or similar. However, the previous video detection methods probably analyze every frame or most of the frames. The same backgrounds of these frames may cause a waste of computing.

The architecture of Context R-CNN [172] is based on Faster R-CNN and the Attention modules. The Attention modules contain Short Term Attention Module and Long Term Attention Module, and the difference between the two kinds of the Attention module is a parameter of the standard dot-product attention formula. Short Term Attention Module and Long Term Attention Module are connected in series.

The method performs well on the Snapshot Serengeti (SS) [173], Caltech Camera Traps (CCT) [174], and CityCam (CC) [175] datasets. Moreover, Context R-CNN performs better than the single frame image detector on the scenes with constant backgrounds.

#### 5.3.14. RetinaNet-VIDeo (RN-VID)

RN-VID [176] is proposed to increase the utilization of the temporal information between adjacent frames, the detection mAP is enhanced on the UA-DETRAC dataset [177] and the UAVDT dataset [178].

The method is based on RetinaNet [44], fuses the feature maps of the adjacent video frames using RetinaNet and VGG-16 [58]. The fusion module re-orders the feature maps, and uses the 1 × 1 convolutional layer as the filter, and the next is to concatenate the subsequent feature maps.

#### 5.3.15. Plug & Play Convolutional Regression Tracker

The proposed network [179] is composed of two Faster R-CNN detectors and one Convolutional Regression Tracking module. The Convolutional Regression Tracking module connects the RPNs of the two Faster R-CNN detectors, to generate the tracklet of an object between video frames.

The Convolutional Regression Tracking module uses a crossed cascade structure. A Region of Interest (RoI) is converted into two branches, the size of the RoI at the bottom branch is 3 times larger than the top branch one, as the object in the top branch RoI may move to the adjacent area. Next, the two branches are crossed concatenated to four channels, and the next is the fully connected layers which are used for the regression.

The Convolutional Regression Tracking module can be inserted into other image detectors, as the module is light weighted. The approach has a good performance on the dataset of ImageNet VID.

#### 5.3.16. Geometry-Aware Spatio-Temporal Network (GAST-Net)

The approach [180] proposed by Xu et al. is generated from Visual Geometry Group, which achieves good results on the datasets of Carla-Vehicle-Pedestrian [181] and DukeMTMC [182] compared with other detectors.

GAST-Net contains two inputs, one is the video frames, which pass through the backbones and fuse into a feature map, the other is the geometry input which is generated from the input video frames. The geometry input passes through Geometry-Aware Attention Maps, and the feature maps are fused with the above feature map, and the next is to pass through the prediction module to classify and locate the objects.

#### 5.3.17. High Quality Object Linking

**Figure 28:**
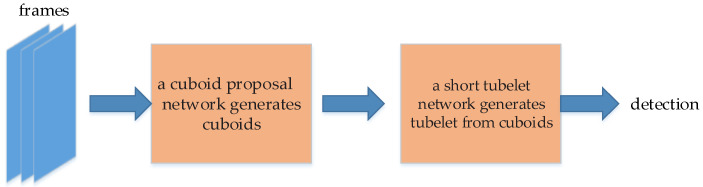
The work flow of High Quality Object Linking.

Tang et al. [183] proposed a tubelet method to connect the same object in adjacent frames. The difference between this method and T-CNN is that it generates tubelets in two stages. In the first stage, a cuboid proposal network is used to generate the cuboid of the object. In the second stage, on the basis of cuboid, different cuboids are connected to generate the tubelet. Thus, the tubelet generated by this method is more accurate, and the video detection accuracy is higher. The work flow of the method is shown in Figure 28.

#### 5.3.18. Spatio-Temporal-Interactive Network (STINet)

**Figure 29:**
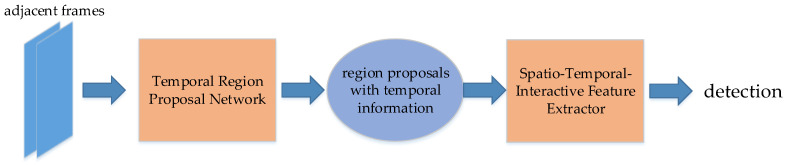
The flow chart of STINet.

Zhang et al. [184] propose a video detection architecture to detect pedestrians. This architecture has two stages: in the first stage, the Temporal Region Proposal Network (T-RPN) is used to extract region proposals from adjacent frames, and these region proposals are connected to generate region proposals with temporal information. In the second stage, Spatio-Temporal-Interactive (STI) Feature Extractor is used to detect these region proposals. This method has achieved state-of-the-art results in pedestrian detection. The flow chart is shown in Figure 29.

#### 5.3.19. Pose-Embedding Network (PEN)

Jiao et al. [185] proposed Pose-Embedding Network (PEN) to detect pedestrians. This method overcomes the problem of part occlusion in video detection, makes the pedestrian detection surpass the state of the art results.

**Figure 30:**
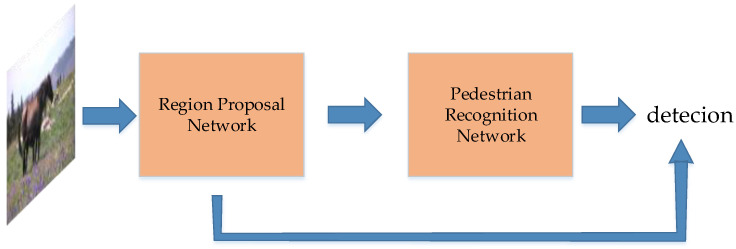
The pipeline of PEN, the final detection is concatenated by Pedestrian Recognition Network and the previous Region Proposal Network.

This method uses human pose information to predict the occluded pedestrian in the next frame. PEN has two steps: the first step is to use the Region Proposal Network (RPN) to extract the features of pedestrians, and to generate the boxes. The second step is to use the Pedestrian Recognition Network to generate human pose information, and perform detection. The pipeline of PEN is shown in Figure 30.

#### 5.3.20. Short-Term Anchor Linking and Long-Term Self-Guided Attention

**Figure 31:**
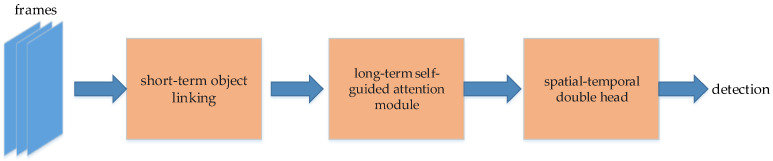
The workflow of Short-Term Anchor Linking and Long-Term Self-Guided Attention.

Cores et al. [186] presents a network architecture which uses the temporal-spatial information in video object detection. The architecture includes 3 components: one is short-term object linking, which integrates the feature maps in the boxes of adjacent frames. One is long-term self-guided attention module, which integrates the feature map of key frames with the feature map of short-term object linking. The final is spatial-temporal double head, which implements the classification and location. The workflow is shown in Figure 31.

#### 5.3.21. Temporal Convolutional Network (TCN)

Kang et al. propose Temporal Convolutional Network (TCN) [187]. TCN operates on tubelet proposals, which are generated by Selective Search (SS) algorithm. TCN generates image object proposals, implements object proposal scoring, realizes high-confidence object proposal tracking among adjacent frames. The method performs object detection around the tubelet box, and replaces the tubelet box with the highest detection score box, thereby reducing the instability of the tubelet box detection.

## 6. Discussion of the Video Object and Human Action Detection Methods

### 6.1. The Performance of the Deep Learning-Based Video Detection Methods

Video detection can be classified into video object detection, video saliency detection and video object behavior detection, etc. Video saliency detection is closely related to video object segmentation. Many valuable deep learning algorithms are developed in the recent years on video detection. YOLO detects the real-time stream videos at a very high frame rate. FGFA [128] can detect blurry videos. CNN has the advantages that traditional methods such as HOG and SIFT do not have, such as translation invariant, robustness, and high detection accuracy. Table 2 summarizes the used datasets and experimental results of one-stage video detection algorithms. Table 3 makes a summarization of those in two-stage video detection algorithms. Table 4 summarizes the video detection algorithms with temporal information of adjacent frames.

The current ideas for improving the speed of video detection mainly are: (A) Increase the single frame detection speed, while maintaining the detection accuracy, and the frame-by-frame method can even reach the real-time detection. (B) Implement the accurate image detection (such as using the two-stage detectors) on the key frames of the video, and skip the non-key frames. Some methods (shown in Section 4) use optical flow or memory history map to transmit feature maps from the non-key frames for detecting. The key points to this approach may be the selection of the key frames and the derivation of the non-key frame feature maps. (C) Another idea is to use the temporal information among the adjacent frames to improve the detection accuracy, such as the LSTM-like approaches, T-CNN [129], etc.

### 6.2. The Evaluation Methods of Video Detection

The video evaluation metrics used in the literatures, such as mAP, accuracy, FLOPs, AP, AR, etc., are almost all calculated by image metrics, i.e., calculated by the frame. Some paper proposes the special video metrics. Mao et al. propose a novel metric called Average Delay to comprehensively evaluate video object detection [188]. Delay refers to the number of frames from when an object appears to when it is detected. Average Delay measures the response time of the detector, which also measures the temporal information of the detector.

Zhu et al. established a dataset for unmanned aerial vehicles (UAVs) video detection named VisDrone, which was taken by UAVs in many cities in China [189]. The dataset is a large-scale benchmark in the field of UAVs. The existing image and video detection algorithms are tested on this dataset.

The methods of video surveillance system using deep learning are as follows: (A) Convolutional Neural Networks (CNN) based methods, the CNN includes AlexNet [190], VGG Net [58], GoogLeNet [45], etc. (B) Restricted Boltzmann Machine (RBM) [191] based methods. RBM is a stochastic neural network, and the related works from RBM are Deep Belief Networks [192], Deep Boltzmann Machines [193] and Deep Energy Models [194]. (C) Auto encoder based methods, which is a kind of unsupervised machine learning methods, and can learn the features of the input data to produce the output data which have the same features with the input data. The usage of the method is Denoising Autoencoder [195] and Contractive Autoencoder [196], etc.

For face detection, the following methods are proposed: (A) Template matching methods, which is based on the pre-learned face templates [197,198]; (B) Feature-based methods, which uses face features [199,200]; (C) Appearance-based methods, which is trained with face data [201,202].

For face recognition, the following methods are proposed: (A) Total matching methods, which compare the whole face region to the pre-learned face database, such as Eigenfaces [203], Principal Component Analysis (PCA) [204], and Linear Discriminant Analysis (LDA) [205]. (B) Feature-based methods, which utilize the specific face features to recognize people, such as the distances, length or shape of mouth, nose and eyes [206]. (C) Hybrid matching methods, which integrate the advantages of the above two methods, especially for the recognition of 3D face images [207].

For face tracking, the following categories of methods are proposed: (A) Point-based tracking, which includes Kalman and Particle filter methods [208]; (B) Multiple Hypothesis Tracking algorithm, which can track multiple objects in the videos [209].

For the image and video quality enhancement, the following algorithms are proposed: (A) Methods based on traditional image enhancement, such as contrast enhancement [210], wavelet based enhancement [211], HDR-based enhancement [212], etc. (B) Context-based video enhancement [213], which utilizes the information of previous frames to increase the brightness of the frame, or to denoise the frame.

The relationships between detection and tracking is that tracking often relies on detection. The main difference between them is that detection makes a classification, tracking only marks the object. Most algorithms show that the faster the detection speed, the lower the detection accuracy, and the lower the detection speed, the higher the detection accuracy.

## 7. Remark of the Limitations and Future Research Directions of Video Detection

The followings are only our own opinions. The limitation of current video object and human action detection algorithms might be that the detection speed and detection accuracy often could not be acquired at the same time. Some algorithms can reach high detection speed while improving the detection accuracy, such as YOLO. However, for those particularly blurry videos, the detection speed could not be increased for now. Most of the current video detection algorithms could not reach the real-time detection speed. On the contrary, the detection and recognition speed might be slow. The phenomenon is especially obvious for those algorithms that use the temporal information. Some literatures of those algorithms did not state the detection speed, but only state the detection metrics. Those particularly blurry videos may require the temporal information detection, thus, we think that real-time detection of the blurry videos could not be implemented for now.

However, the detection accuracy of those algorithms has already been highly improved. Some algorithms can reach a high level on the blurry videos, such as FGFA (shown in Section 5.2.3). Nevertheless, we still think that the video detection speed is important either.

The conceivable future research directions might be the following three aspects: (A) Enhancing the detection accuracy, especially for the blurry videos. The main idea is to use the temporal information among adjacent frames. (B) Enhancing the detection speed. The existing algorithms have advantage on those distinct videos, however, for those blurry videos, the detection speed could be improved. (C) Enhancing the detection accuracy and speed simultaneously. The main idea might be extracting the key frames. Since detecting the key frames only, the detection speed could be enhanced. The key frames are often clearer, and have more standard shape objects or human action shapes, which could enhance the detection accuracy and speed both.

## 8. Conclusions

The discussed algorithms in the paper include object detection, action analysis, etc. The video detection algorithms are all frame-based for now. Currently there are three ideas for the video detection: (A) the first is to detect each frame. Some algorithms, such as YOLO, can realize very fast detection speed; (B) the second is to extract the key frames, and the detection depends on the algorithm of extracting key frames; (C) the third is to use LSTM structure or the optical flow method for extracting the temporal information among adjacent frames. The image detector can be applied to video detection in the three ideas above. Some video detection algorithms extract the temporal information by tracking on adjacent frames, such as the bounding box tracklet of the same object in adjacent frames [129]. Many video detection algorithms are based on image detection algorithms, and the video detection metrics in these algorithms use image detection metrics by frames.

For blurry videos, or the videos with part occlusion, making full use of the temporal information among adjacent frames can significantly improve the detection accuracy, since the blurry or occluded objects could be detected in adjacent frames. From the existing literatures, the approaches of using the temporal information of adjacent frames include the LSTM structure, the optical flow method and convolution among adjacent frames. Some approaches transfer feature maps between frames via convolutions, such as Motion History Image. We think extracting the key frames could enhance both the video detection accuracy and speed.

## Figures and Tables

**Table 1:** The structure of DarkNet19.

	Dimension	Convolution Kernel	Stride	Output
Conv.	32	3 × 3		224 × 224
Maxpool		2 × 2	2	112 × 112
Conv.	64	3 × 3		112 × 112
Maxpool		2 × 2	2	56 × 56
Conv.	128	3 × 3		56 × 56
Conv.	64	1 × 1		56 × 56
Conv.	128	3 × 3		56 × 56
Maxpool		2 × 2	2	28 × 28
Conv.	256	3 × 3		28 × 28
Conv.	128	1 × 1		28 × 28
Conv.	256	3 × 3		28 × 28
Maxpool		2 × 2	2	14 × 14
Conv.	512	3 × 3		14 × 14
Conv.	256	1 × 1		14 × 14
Conv.	512	3 × 3		14 × 14
Conv.	256	1 × 1		14 × 14
Conv.	512	3 × 3		14 × 14
Maxpool		2 × 2	2	7 × 7
Conv.	1024	3 × 3		7 × 7
Conv.	512	1 × 1		7 × 7
Conv.	1024	3 × 3		7 × 7
Conv.	512	1 × 1		7 × 7
Conv.	1024	3 × 3		7 × 7
Conv.	1000	1 × 1		7 × 7
Averagepool		Global		1000
Softmax				

**Table 2:** The state-of-the-art one-stage video detection algorithms. The image detection algorithms can be used to detect videos by the way of frames. FPS denotes frame per second.

Algorithm	Category	Dataset	Results
AlexNet	image detection	2012 ImageNet Classification Challenge	Champion
FCN	image segmentation	VOC2011, VOC2012	mean IU: more than 62.0%
YOLOv1	video detection	VOC2007 and 2012	mAP: 63.4%, FPS: 45
YOLOv2	video detection	VOC2007 and 2012	mAP: 78.6%, FPS: 40
YOLOv3	video detection	COCO	mAP-50: 57.9%, Inference time: 51 ms
SSD	image detection	VOC2007	mAP: 72.1%, FPS: 58
DSSD	image detection	VOC2007 and 2012	mAP: more than 80.0%
RSSD	image detection	VOC2007 and 2012	mAP: 80.8%
FSSD	image detection	VOC2007 and 2012	mAP: 84.5%, FPS: 35.7
FPN	image detection	COCO mini-val set	AP at 0.5 IOU: 56.9%

**Table 3:** The state-of-the-art two-stage image detection algorithms, which can be used to detect videos by frames.

Algorithm	Dataset	Results	Note
R-CNN	VOC2007	mAP: 66%	
SPP Net	VOC2007, ILSVRC 2014	mAP: 60.9%(VOC), 2nd(ILSVRC)	Input image of any size
Fast R-CNN	VOC 2007 and 2012	mAP: 70%	Training and testing time reduced
Faster R-CNN	VOC 2007 and 2012	mAP: 73.2%	Input image of any size
ResNet-101	COCO VOC 2007 and 2012 ILSVRC 2015	mAP: 48.4% mAP: 76.4% champion	
GoogLeNet	ILSVRC 2014 ImageNet	champion Top-5 error: 3.8%(Inception-v4)	
Mask R-CNN	COCO	50% IoU Keypoint, AP: 87.3%	

**Table 4:** The state-of-the-art video detection algorithms, which operate on multiple adjacent frames. ILSVRC denotes the ImageNet Large-Scale Visual Recognition Challenge.

Algorithm	Dataset	Results
3D Convolution	TRECVID KTH	AP: 0.7137 accuracy: 90.2%
T-CNN (Kang et al.)	ILSVRC2015	Champion
TCN (Bai et al.)	Sequential MNIST Permuted MNIST	accuracy: 99% accuracy: 97.2%
DFF	ImageNet VID Cityscapes	mAP: 73.1% mIoU: 69.2%
FGFA	ImageNet VID	mAP: 83.5%
Association LSTM	Youtube-Objects	mAP: 72.14%
STMN	ImageNet VID	mAP: 80.5%
MANet	ImageNet VID	mAP: 86.9%
D&T	ImageNet VID	mAP: 79.8%
ST-Lattice	ImageNet VID	mAP: 79.0%, FPS: 62
TD-Graph LSTM	Charades	mAP: 19.52%
STSN	ImageNet VID	mAP: 80.4%
Patchwork	ImageNet VID	mAP: 58.7%
PSLA	ImageNet VID	mAP: 81.4%
RDN	ImageNet VID	mAP: 84.7%
LSTS	ImageNet VID	mAP: 82.1%
SELSA	ImageNet VID	mAP: 86.91%
MEGA	ImageNet VID	mAP: 85.4%
TSM	Kinetics UCF101 HMDB51 Something-Something ImageNet VID	accuracy: 74.1% accuracy: 95.9% accuracy: 73.5% accuracy: 47.3% mAP: 83.4%

## Data Availability

No new data were created in this study. Data sharing is not applicable to this article.
